# Proteomic Stable Isotope Probing Reveals Biosynthesis Dynamics of Slow Growing Methane Based Microbial Communities

**DOI:** 10.3389/fmicb.2016.00563

**Published:** 2016-04-29

**Authors:** Jeffrey J. Marlow, Connor T. Skennerton, Zhou Li, Karuna Chourey, Robert L. Hettich, Chongle Pan, Victoria J. Orphan

**Affiliations:** ^1^Division of Geological and Planetary Sciences, California Institute of TechnologyPasadena, CA, USA; ^2^Chemical Sciences Division, Oak Ridge National LaboratoryOak Ridge, TN, USA

**Keywords:** proteomics, stable isotope probing, methane seeps, anaerobic oxidation of methane, environmental microbiology

## Abstract

Marine methane seep habitats represent an important control on the global flux of methane. Nucleotide-based meta-omics studies outline community-wide metabolic potential, but expression patterns of environmentally relevant proteins are poorly characterized. Proteomic stable isotope probing (proteomic SIP) provides additional information by characterizing phylogenetically specific, functionally relevant activity in mixed microbial communities, offering enhanced detection through system-wide product integration. Here we applied proteomic SIP to ^15^${\text{NH}}_{4}^{+}$ and CH_4_ amended seep sediment microcosms in an attempt to track protein synthesis of slow-growing, low-energy microbial systems. Across all samples, 3495 unique proteins were identified, 11% of which were ^15^N-labeled. Consistent with the dominant anaerobic oxidation of methane (AOM) activity commonly observed in anoxic seep sediments, proteins associated with sulfate reduction and reverse methanogenesis—including the ANME-2 associated methylenetetrahydromethanopterin reductase (Mer)—were all observed to be actively synthesized (^15^N-enriched). Conversely, proteins affiliated with putative aerobic sulfur-oxidizing epsilon- and gammaproteobacteria showed a marked decrease over time in our anoxic sediment incubations. The abundance and phylogenetic range of ^15^N-enriched methyl-coenzyme M reductase (Mcr) orthologs, many of which exhibited novel post-translational modifications, suggests that seep sediments provide niches for multiple organisms performing analogous metabolisms. In addition, 26 proteins of unknown function were consistently detected and actively expressed under conditions supporting AOM, suggesting that they play important roles in methane seep ecosystems. Stable isotope probing in environmental proteomics experiments provides a mechanism to determine protein durability and evaluate lineage-specific responses in complex microbial communities placed under environmentally relevant conditions. Our work here demonstrates the active synthesis of a metabolically specific minority of enzymes, revealing the surprising longevity of most proteins over the course of an extended incubation experiment in an established, slow-growing, methane-impacted environmental system.

## Introduction

Marine methane seep sediments harbor complex microbial communities that play significant roles in the global carbon and sulfur biogeochemical cycles (Jørgensen and Kasten, 2006; Reeburgh, 2007). Studies of these features have predominately focused on biodiversity (e.g., Bidle et al., 1999; Knittel et al., 2005; Pernthaler et al., 2008), the energetic and biochemical basis of syntrophic partnerships (Alperin and Hoehler, 2009; Stams and Plugge, 2009), and ecosystem-wide contributions to global methane processing (Reeburgh, 2007). Such studies revealed that one of the dominant metabolisms at seep sites—the anaerobic oxidation of methane (AOM)—is enacted by consortia of anaerobic methanotrophs (ANME) most closely related to methanogens and sulfate-reducing deltaproteobacteria. Phylogenetically-linked measures of anabolic activity and growth of AOM consortia using stable isotope probing have identified intricately coupled microbial metabolisms (Krüger et al., 2008a; Dekas et al., 2009; Orphan et al., 2009), while metagenomics and metatranscriptomics studies have pointed to metabolic potential and associated biochemical pathways (Hallam et al., 2004; Pernthaler et al., 2008; Meyerdierks et al., 2010; Stokke et al., 2012; Wang et al., 2014). Proteomic stable isotope probing (SIP) couples these experimental objectives by offering a functionally- and phylogenetically-constrained enzymatic profile of constituent organisms as well as a temporal reporter of protein synthesis and metabolic response to distinct conditions (Pan et al., 2011; Seifert et al., 2012; Justice et al., 2014; Mohr et al., 2014).

In environments such as anoxic methane seep sediment and an array of subsurface habitats, where energy can become limiting, microbes frequently exhibit extremely slow growth rates and are particularly recalcitrant to activity-based analyses. Radiotracers are sensitive probes to rates on the order of 10^−19^–10^−16^ mol cell^−1^ day^−1^ (Parkes et al., 1990), but the range of discoverable metabolic reactions is severely constrained. Fluorescence *in situ* hybridization (FISH) coupled with nanoscale secondary ion mass spectrometry (nanoSIMS; e.g., Wagner, 2009) can detect assimilation rates as low as 10^−17^ mol cell^−1^ day^−1^ (Morono et al., 2014) and visualize phylogenetically constrained spatial associations, though experimental throughput is low and only assimilatory processes can be queried. Whole-cell bioorthogonal non-canonical amino acid tagging (BONCAT) coupled with FISH can be used to fluorescently visualize microorganisms active in protein synthesis (Hatzenpichler et al., 2014), but identification of specific newly synthesized proteins has only been successfully applied in a few cases (Babin et al., 2016) and requires further development for complex environmental systems. Proteomic SIP represents an important entry in the analysis of metabolic activity in low-energy microbial systems, due to its spatially broad, yet functionally- and phylogenetically-specific search space. The procedure is able to identify particular metabolic pathways or enzyme-mediated responses (Bozinovski et al., 2012; Justice et al., 2014) that can be integrated across constituents of a particular lineage, offering an opportunity to access a segment of the low-activity biosphere that might go undetected by other methods due to low levels of anabolism by individual organisms. Although challenges remain—particularly surrounding protein extraction, peptide quantification, database collation, and the interpretation of non-detections—proteomic SIP offers a promising method for assessing the *in vivo* activity and catalytic function of microbial communities.

Culture-independent studies of energy-limited methane seep settings have included meta-omics investigations focused largely on the pathway responsible for AOM. These investigations have revealed that AOM likely utilizes the same enzymes as methanogenesis, operating in the reverse direction (Hallam et al., 2004; Meyerdierks et al., 2010). ANME-1 draft genomes and fosmids, however, lack the *mer* gene (Meyerdierks et al., 2010; Stokke et al., 2012), prompting the proposition of a reverse-methanogenesis bypass (Meyerdierks et al., 2010). ANME-2 lineages, including ANME-2a (Wang et al., 2014), and nitrate-reducing ANME-2d (Haroon et al., 2013) genomes, as well as a magnetic enrichment of ANME-2c consortia (Pernthaler et al., 2008), contained the *mer* gene. Corresponding gene expression profiles revealed ANME-1 methylenetetrahydromethanopterin dehydrogenase (*mtd*), heterodisulfide reductase subunits A and B (*hdrAB*), and methyl-coenzyme M reductase subunit A (*mcrA*) transcripts (Meyerdierks et al., 2010). ANME-2a and ANME-2d transcriptomic datasets exhibited highly expressed methyl-tetrahydromethanopterin coenzyme M methyltransferase (*mtr*), *mcr, mer*, and methenyltetrahydromethanopterin cyclohydrolase (*mch*) and substantially lower levels of *mtd* and several formylmethanofuran dehydrogenase (*fmd*) subunits (Haroon et al., 2013; Wang et al., 2014).

Proteomic SIP in environmental samples enables the identification of isotopically enriched proteins synthesized by a microbial assemblage after incubation with an isotopically labeled substrate (e.g., ^15^N-ammonium). The application of proteomic SIP to slow growing methane seep microbial communities offers an opportunity to examine production patterns of reverse methanogenesis enzymes central to ANME-facilitated methane-oxidation, as well as to identify the proteins expressed by syntrophic bacterial partners and other organisms within the seep ecosystem. This approach can highlight metabolic shifts resulting from incubation conditions, reveal the fraction of proteins that are newly synthesized, and potentially facilitate the construction of a framework for interspecies metabolic relationships (Jehmlich et al., 2008; Hawley et al., 2014). The greatest insights from environmental metaproteomic studies to date have been associated with time-resolved analyses of low-complexity microbial systems (i.e., acid mine drainage microbial biofilms; Denef et al., 2010; Pan et al., 2011), while similar efforts with sponge symbionts (Liu et al., 2012), sinking marine particulates (Moore et al., 2012), and the human gut (Verberkmoes et al., 2009; Xiong et al., 2015) have clarified interspecies relationships and dominant biogeochemical functionalities. There have been comparatively few studies applying proteomics to deep-sea methane seep ecosystems; these have focused on individual enzymes (Krüger et al., 2003), explored specific biochemical questions (Glass et al., 2014), or achieved relatively low coverage of the full ecosystem (Stokke et al., 2012). Here we use proteomics and proteomic SIP to examine multiple methane seep sediment incubations containing diverse ANME and bacterial lineages and exhibiting a range of methanotrophic activity levels.

## Methods

### Sample collection from methane seeps

Seafloor sediment was collected from active methane seeps at Hydrate Ridge, Oregon, an area that has served as a natural laboratory for the study of AOM for over a decade (Suess et al., 1999; Treude et al., 2003; Boetius and Suess, 2004; Marlow et al., 2014a). At Hydrate Ridge, the sulfate-methane transition zone occurs in the top several centimeters of seafloor sediment; within this horizon, microbial aggregate abundance, methane oxidation rates, and sulfate reduction rates are highest (Boetius et al., 2000; Boetius and Suess, 2004). Methane concentrations at active seep sediments have been measured and modeled at values up to 70 mM (Boetius and Suess, 2004) and 50 mM (Tryon et al., 2002), respectively; sulfate concentrations decrease with sediment depth, concomitant with augmented sulfide levels up to 26 mM (Sahling et al., 2002). Samples for this study were collected during two field campaigns, including R/V *Atlantis* leg AT-15-68 using the *DSV Alvin* (August 2010) and *Atlantis* leg AT-18-10 using the *ROV Jason* II (September 2011). Samples are referenced according to unique four-digit serial numbers. Push core (PC) 16 from *Alvin* dive 4635 at Hydrate Ridge South (44° 34.20′ N, 125° 8.87′ W, 775 m depth) was collected from a microbial mat indicative of active methane seepage. The recovered 12 cm of sediment from PC-16 was divided into the upper 0-6 cm (sample #3730, used for metagenomic sequencing) and the lower 6-12 cm (#3731, used for metaproteomic and nanoSIMS-based analyses). #5133 and #5579 refer to elevator 3A push cores 47 and 41, respectively, collected at Hydrate Ridge North during *Jason* II dive J2-593 from an area of active seepage marked by bubble ebullition and surface expression of white microbial mat (44° 40.17′ N, 125° 5.89′ W, 600 m depth). #5133 recovered 9 cm of sediment, with a dark sulfidic zone at ~4-5 cm depth; #5579 contained 12 cm of sediment.

Shipboard, push cores were immediately transferred to a 4°C walk-in cold room and processed within several hours. To prepare sediment for future experimentation, compacted sediment was stored in anoxic, Ar-flushed mylar bags at 4°C until use. Incubations prepared for metaproteomic analysis were amended with 1 mM of ^14^${\text{NH}}_{4}^{+}$ or ^15^${\text{NH}}_{4}^{+}$. Inhibition of microbial activity due to ^15^${\text{NH}}_{4}^{+}$ was unlikely, as methanogen and sulfate reducing bacterial pure cultures, as well as methane seep sediment, showed no adverse effects when presented with ^15^N ammonia ratios up to 100% (Krüger et al., 2008b).

### Laboratory-based ^15^N-SIP incubations

**Figure 1:**
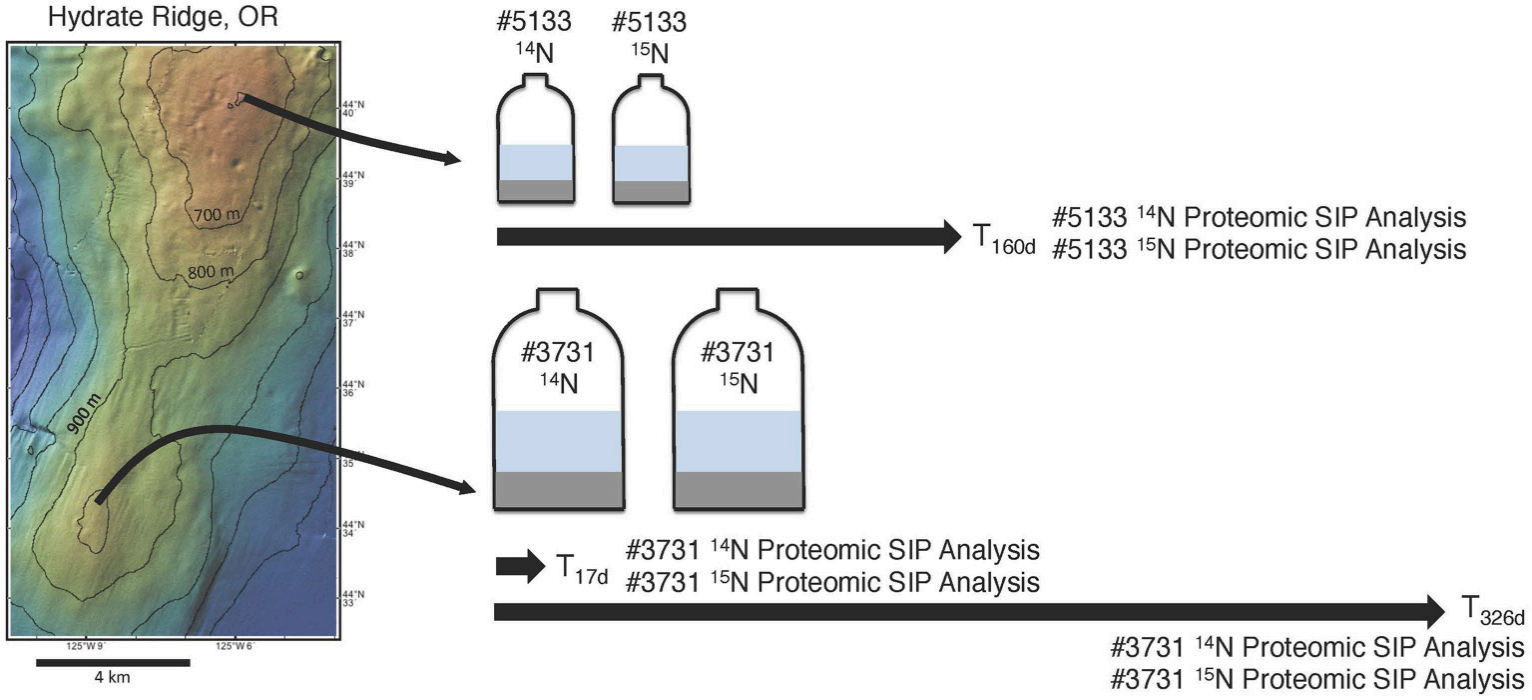
**A schematic diagram of the proteomic SIP sampling approach used in this study**. Sediment was collected from active seep areas at Hydrate Ridge North for incubation #5133 and Hydrate Ridge South for #3731 (see text for full sampling details). Sediment from both sample sites was used as inoculum in parallel methane-infused incubations, with one bottle from each set receiving 1 mM ^14^NH_4_Cl and the other receiving 1 mM ^15^NH_4_Cl. Both #3731 incubations were sampled for proteomic SIP after 17 and 326 days; both #5133 incubations were sampled for proteomic SIP after 160 days.

In advance of experimental set-up, separate sediment slurries from #3731 and #5133 were reconstituted under anoxic conditions using 0.22 μm filtered, anoxic N_2_-sparged Hydrate Ridge bottom water (at a 1:2 sediment:bottom water ratio by volume) and these mesocosm incubations (1L bottle) were maintained with a 2 bar CH_4_ headspace for 1 month. The ${\text{NH}}_{4}^{+}$ concentration of the filtered bottom water was 496 μM, as measured with a Dionex DX-500 Ion Chromatograph using the protocol specified in Green-Saxena et al. (2014). Incubations for proteomic SIP were collected from this mesocosm experiment; four incubations were set up, corresponding to two sediment samples (#3731 and #5133), each receiving two experimental treatments (Figure 1). 60 mL of the #3731 slurry was transferred into each of two sterile 120 mL serum bottles in an anaerobic chamber and supplemented with 1 mM final concentration of either ^14^NH_4_Cl or ^15^NH_4_Cl (99% enrichment, Cambridge Isotope Laboratories, Inc., Andover, MA). The vials were sealed with butyl rubber stoppers, flushed for 5 min each with Ar and then CH_4_, and overpressured with CH_4_ to a final pressure of 2 bars. #5133 ^14^${\text{NH}}_{4}^{+}$ and ^15^${\text{NH}}_{4}^{+}$ experiments were set up in a similar manner, though the size of the serum bottles was 35 mL and initial slurry volumes were 20 mL. All samples were kept at 4°C in the dark during the incubation period.

Aggregate counts were conducted at incubation start and end points, and sulfide concentrations were determined at four time points. To quantify the number of cell aggregates, incubation sediment was subjected to a percoll density separation performed according to Orphan et al. (2009). Sediment slurry (~60 μL) was mixed with 1290 μL of TE (pH = 9) and heated for 3 min at 60°C. The sample tubes were put on ice for 5 min; 4.5 μL of 30% H_2_O_2_ was then added (to deactivate peroxidase) and incubated at room temperature for 10 min. Tubes were put back on ice, and 150 μL sodium pyrophosphate was introduced. Sample mixtures were sonicated on ice 3 times at 8 W (Branson sonifier 150) for 10 s each time and applied to the density gradient vials. The vials were then centrifuged at 4800 rpm for 15 min at 4°C. The supernatant was filtered through both 3 μm and 0.22 μm polycarbonate filters, which were then rinsed with 2 mL PBS and dehydrated with 2 mL 1:1 ethanol:PBS. Aggregate counts were performed under epifluorescence microscopy (60x objective lens, Olympus BX51 microscope, Olympus, Melville, NY) using DAPI; the corresponding morphologies have been shown via FISH analysis to consist dominantly of ANME-SRB aggregates active in anaerobic methane oxidation (Orphan et al., 2002, 2009; Marlow et al., 2014b). Sulfide concentrations were determined using the Cline Assay (Cline, 1969) as described in Dekas et al. (2009).

### 16S rRNA gene tag sequencing

DNA from ^14^N incubation samples #3731 T_0*d*_, #3731 T_326*d*_, and #5133 T_170*d*_ was extracted from ~0.5 g of sediment using the UltraClean Soil DNA isolation kit (Mo Bio Laboratories, Carlsbad, CA) and microbial community composition was profiled using the 515f (5′-GTGYCAGCMGCCGCGGTAA-3′) and 806r (5′-GGACTACNVGGGTWTCTAAT-3′) primer pair (Caporaso et al., 2011). PCR barcoding reactions were conducted in house at Caltech and amplicons were outsourced to Laragen Inc. (Laragen Inc., Culver City, CA) for sequencing on the Illumina MiSeq platform. Resulting sequence data consisted of 250 bp paired end reads, which were assembled into a single contig and analyzed using Qiime 1.8 (Caporaso et al., 2010). Sequences were trimmed to a minimum quality value of 30, and taxonomy was assigned to each OTU representative based on the SILVA 115 rRNA database uclust (Edgar et al., 2011) using a sequence similarity threshold of 99%. The full protocol is contained in a GNU makefile (Supplemental Data Sheet 1); the makefile must be in the same directory as the raw sequencing reads, and qiime 1.8 and usearch must be installed. The complete protocol can be run by typing “./orphanlab_itag_protocol.mk all” into the command prompt.

### Metagenome database construction and annotation

The metagenomic database against which tandem mass spectra were referenced to identify proteins was constructed from several sources (Table 1). DNA was extracted using the UltraClean Soil DNA isolation kit (Mo Bio Laboratories, Carlsbad, CA) from ~0.5 g of bulk methane seep sediment (before distribution to the proteomic SIP incubations) and from magneto-FISH capture experiments from Hydrate Ridge (Pernthaler et al., 2008; Dekas et al., 2009; Glass et al., 2014; Table 1). Bulk sediment metagenomes from samples #3730, #5133, and #5579 were sequenced using the Illumina HiSeq platform; four magneto-FISH metagenomes (targeting ANME-2c) were sequenced using 454 GS-20 (BC02, Pernthaler et al., 2008), 454 GS-FLX (BC03), and 454 GS-Titanium (BC04 and BC05). Metagenomes derived from Illumina HiSeq data were processed as follows. Raw sequencing reads were quality trimmed (using a threshold of 0.05) and assembled using CLCBio genomics workbench 6.0. Sequencing reads from each metagenome were assembled individually and two assemblies were performed by combining sequencing reads from the three datasets. For each assembly, the raw sequencing reads from all datasets were aligned to contigs >500 bp with BWA 0.7.5a (Li, 2013) using the “mem” algorithm and converted into bam files using samtools 0.1.19 (Li et al., 2009). These bam files were used as input for GroopM 0.2 (Imelfort et al., 2014), which generated genome bins. The “refine” command in GroopM was used to improve the genome bins manually by removing contigs with unusual coverage or tetramer frequencies. The completeness and contamination of genome bins was determined using CheckM 0.9.4 (Parks et al., 2014).

**Table 1:** **Details of the constituents that comprise the metagenomic database used in this study**.

**IN-HOUSE METAGENOMES**			
**Metagenome name**	**Source material**	**Sequencing platform**	**# of ORFs**
BC	Bead Capture from Hydrate Ridge Seep Sediment, E3A PC47, 0–9 cm	454	25208
V01	Hydrate Ridge Seep Sediment, AD 4635 PC16, 0–6 cm	Illumina	4599
V02	Hydrate Ridge Seep Sediment, E3A PC47, 0–9 cm	Illumina	4841
3730	Hydrate Ridge Seep Sediment, AD 4635 PC16, 0–6 cm	Illumina	118384
5133	Hydrate Ridge Seep Sediment, E3A PC47, 0–9 cm	Illumina	78724
5579	Hydrate Ridge Seep Sediment, E3A PC41, 0–12 cm	Illumina	133690
3datasets	Combined assembly of 3730, 5133, 5579		133255
5datasets	Combined assembly of 3730, 5133, 5579, VO1, VO2		246534
**OTHER SEQUENCE DATA**			
**Name**	**Source material**	**Sequencing platform**	**# of ORFs**
Hallam Fosmids^a^	Eel River Basin Seep Sediment	Sanger	2219
Meyerdierks Fosmids^b^	Black Sea Microbial Mats	Sanger	3122
ANME-2a^c^	Capt Aryutinov Mud Volcano	Illumina	4031
ANME-2d^d^	Sediment/Wastewater Sludge Enrichment	Illumina	3427
Cultured Organisms	NCBI Genome Repository		591976

References correspond to

a Hallam et al. (2004).

b Meyerdierks et al., 2010.

c Wang et al., 2014.

d Haroon et al., 2013.

*The list of cultured organisms whose genomes were included in the metagenome can be found in Supplemental Data Sheet 2*.

Bead capture metagenomes derived from 454 sequencing platforms generated ~25–145 Mb of sequence data per sample. Sequencing reads from all bead capture metagenomes were combined and assembled using Newbler. Open reading frames (ORFs) were called using prodigal 2.5 (Hyatt et al., 2010) using the metagenome mode (command line ‘prodigal –i < FILE> -p meta –a < translation.faa> -o < output.gbk>’). Only ORFs that were considered complete (contained both a start and stop codon) were considered for inclusion to the metagenomic database. This data set was supplemented with fosmid sequence from seep sediment and physiologically relevant genomes from cultured organisms (Supplemental Data Sheet 2). All ORFs were clustered at 100% identity using uclust (Edgar et al., 2011) to remove duplicates.

Functional annotations were derived from previously determined NCBI annotations (for cultured organism genomes) or the KEGG Automatic Annotation Server (KAAS; Moriya et al., 2007) for genes from the metagenomes. For cultured organisms, phylogenic affiliations of gene products were incorporated from NCBI annotations. For the metagenomes, the phylogeny of genome bins with low (< 10%) contamination was assessed using the CheckM parsimonious genome tree, and the proteins derived from that genome bin were all given the same taxonomy. For all other proteins derived from the metagenomes, individual genes were compared to the NCBI nr database using blastp 2.2.29+ (Camacho et al., 2009) and taxonomy was determined using the lowest common ancestor algorithm implemented by Blast2lca 0.600 (https://github.com/emepyc/Blast2lca). #5133 ^15^N enriched proteins that were annotated as “hypothetical proteins” by KAAS (*n* = 110) were subjected to a secondary search process. Corresponding ORFs were blasted against UniRef 90 (Suzek et al., 2007) using the UniProt webserver (UniProt Consortium, 2008) and an e-value cutoff of 10^−30^. Annotations derived from this process were incorporated into the data analysis, while ORFs that remained “hypothetical” or “uncharacterized” were searched by hand against the Interpro database (Mitchell et al., 2015) for putative functional domains. The subset of these proteins that was also present in all six proteomes is discussed below.

Proteins involved in the reverse methanogenesis pathway were resolved to finer phylogenetic resolution to determine their ANME subtype affiliation. Proteins from the metagenomes were combined with proteins downloaded from the KEGG database and aligned using muscle 3.8.31 (Edgar, 2004). A phylogenetic tree was built from the alignment using FastTree 2.1.7 (Price et al., 2010) and visualized with Archaeopteryx 0.9901 (https://sites.google.com/site/cmzmasek/home/software/archaeopteryx). ANME subtypes were determined by identifying monophyletic groups with known ANME-1, ANME-2a, ANME-2b, and ANME-2c sequences and cross validating between genes from the same contigs to resolve inconsistencies in phylogenetic placement. Protein alignments were visualized in Jalview 2.8.2 (Waterhouse et al., 2009).

### Metaproteomic sample processing

The proteomic SIP sampling approach is shown in Figure 1. Sample #3731 incubations were sampled after 17 days (T_17*d*_) and 326 days (T_326*d*_) of incubation with methane; #5133 was sampled after 160 days (T_160d_). These sampling times were determined based on the multi-month doubling times of ANME-SRB aggregates (Orphan et al., 2009), and the lack of significant ^15^N enrichment in the T_17*d*_ protein sample that necessitated the extended incubation timescale. At the designated sampling times, 20 mL (#3731) or 5 mL (#5133, small volume due to sample limitation) of sediment slurry was removed and immediately flash frozen with liquid nitrogen to stop biological reactions and preserve proteins representative of the incubation conditions. Proteins were extracted and digested using an SDS-TCA-based protocol as outlined in Chourey et al. (2010). To summarize, frozen sediment slurry was thawed, and 10 g (#3731 T_17d_ and T_326d_) or 5 g (#5133) of wet sediment was suspended in 20 mL of lysis buffer [5% SDS, 50 mM Tris-HCl, pH 8.5, 0.15 M NaCl, 0.1 mM EDTA, 1 mM MgCl_2_, 75 mM dithiothretiol (DTT)]. The mixture was vortexed and immersed in a boiling water bath for 20 min, allowed to cool for 5 min, and transferred to a sterile 50 mL Falcon tube. A 10-min centrifugation at 2500 × g separated soil particles and cellular debris from the whole cell lysate, which was retained in the supernatant. The supernatant was pipetted into sterile 2-mL Eppendorf tubes, amended with 100% trichloroacetic acid (TCA) to a final concentration of 25%, vortexed, and stored overnight at –20°C to precipitate the extracted proteins. Protein pellet was collected by centrifugation at 21,000 g for 20 min, and the supernatant was discarded. The protein pellet was washed with chilled acetone followed by centrifugation at 21,000 g for 5 min. After three acetone washes, the protein pellet was air dried and solubilized in Guanidine buffer [6 M guanidine HCl, 10 mM DTT in Tris buffer (50 mM Tris with 10 mM CaCl_2_, pH 7.8)] followed by an overnight incubation at 37°C. The mixture was diluted six-fold with Tris CaCl_2_ buffer to dilute the guanidine concentration to 1 M and avoid trypsin deactivation. Trypsin was added at a predetermined concentration of 40 μg trypsin/1–3 mg total protein in two separate doses of 20 μg each, separated by several hours of incubation at 37°C; the second addition was allowed to incubate overnight. DTT was again added to a final concentration of 10 mM and allowed to incubate for 45 min at room temperature to linearize the peptides. The peptide solution was de-salted using a C_18_+ Sep-Pak solid phase extraction cartridges (Waters, Milford, MA) solvent exchanged into acidified water (HPLC grade water, 0.1% formic acid) using a Savant SpeedVac (ThermoFisher Scientific, Waltham, MA), filtered through an Ultrafree-MC centrifugal filter device (Millipore, Billerica, MA), and stored at −80°C until two-dimensional LC−MS/MS analysis.

Duplicate runs of LC-MS/MS analysis were conducted for each sample as described previously (Pan et al., 2011; Justice et al., 2014). Peptides (~75 μg) were pressure-loaded onto a split-phase Reverse Phase (RP)-SCX column, which was aligned with an in house RP-packed PicoFrit tip (New Objective, Woburn, MA). Peptides were eluted and chromatographically separated via a 24-h Multi-Dimensional Protein Identification Technology (MuDPIT), using an 12-step salt gradient using liquid chromatography (LC) as described earlier (Sharma et al., 2012). Peptide separation, fragmentation, and measurements were carried out using a range of Orbitrap instruments in keeping with the rapid development of the field's best available technology: #3731 ^14^N T_17d_ and #3731 ^15^N T_17d_ were analyzed on an LTQ Orbitrap Velos, #3731 ^14^N T_326d_ on an LTQ Velos, #3731 ^15^N T_326d_ on an LTQ Orbitrap Velos Pro, and #5133 ^14^N T_160d_ and #5133 ^15^N T_160d_ on an LTQ Orbitrap Elite. The mass spectrometers (Thermo Fisher Scientific, Germany) were coupled to the Ultimate 3000 HPLC system (Dionex, USA) and operated in data dependent mode using the Thermo Xcalibur software V2.1.0. Each full scan was followed by individual MS/MS scans of the 20 most abundant parent ions, which were fragmented via collision-activated dissociation (CID) using 35% collision energy, a mass exclusion width of 0.2 m/z, and a dynamic exclusion duration of 60 s.

### Protein search parameters

MS/MS data was searched using the Sipros/ProRata 3.0 program (Wang et al., 2013); precursor mass tolerance and fragment ion mass tolerance were 0.05 Da and 0.02 Da for the data generated on Orbitrap instruments and 3 Da and 0.5 Da for those generated on LTQ Velos, respectively. The metaproteomic database was predicted from the metagenomic database (Table 1) and concatenated with its reverse sequence to estimate its false positive detection rate (FDR; Elias and Gygi, 2007). The search results were filtered to achieve a 1% FDR at the peptide level, and proteins were inferred from the identified peptides using parsimony rules (Nesvizhskii and Aebersold, 2005). The most stringent settings, which have been used in several proteomics studies (Denef et al., 2009; Verberkmoes et al., 2009; Liu et al., 2012), are a 1% FDR, 2 total peptides, and 1 unique peptide (expressed with the notation [1%, 2TP, 1UP]). However, the [1%, 1TP, 1UP] approach has been promoted on statistical grounds (Gupta and Pevzner, 2009) and used in several environmental and culture-based proteomics studies (Ram et al., 2005; Dong et al., 2010; Webb et al., 2013; Hebert et al., 2014). With the acknowledgement that less stringent protein identification parameters result in lower confidence of a protein's presence, we present both [1%, 1TP, 1UP] and [1%, 2TP, 1UP] results, clearly noting the provenance of each identification.

The following PTMs were dynamically searched in McrA analysis: hydroxylation of proline and lysine; monomethylation of arginine, lysine, aspartic acid, and glutamic acid; dimethylation of arginine and lysine; trimethylation of lysine; phosphorylation of serine, threonine, tyrosine, histidine and aspartic acid; acetylation of lysine; S-nitrosylation of cysteine; nitration of tyrosine; and methylthiolation of aspartic acid. The raw search results from both the PTM search and the broader search against the full database were filtered together to achieve a 1% FDR at the peptide level, allowing the top peptide of a MS/MS spectrum from the PTM search to compete with the top peptide of the same spectrum from the regular search. PTMs were localized based on the DeltaP score as described in Li et al. (2014).

### SIP enrichment threshold and analysis

A smaller database constructed of all ORFs identified during the initial non-SIP 1TP, 1UP analyses (22,312 ORFs) and their reverse decoy sequences was used for SIP searches and filtering due to computational limitations. Because protein identification is predicated on the detection of constituent peptides, and because 1TP, 1UP identifications were considered in the analysis, designations of “enriched” proteins (defined as proteins that contain ^15^N with statistical confidence) must be made at the peptide level.

Procedural steps for determining ^15^N atom% are fully described elsewhere (Pan et al., 2011; Fischer et al., 2013; Justice et al., 2014). Briefly, for each peptide sequence from *in silico* digestion of target/reverse proteins in the database, the most abundant mass and isotopic distribution of b and y ions were predicted at a given ^15^N atom% ranging from 0 to 100% (1% intervals), assuming uniform ^15^N enrichment. For each experimental MS/MS spectrum, candidate peptides were found if their most abundant masses at a given ^15^N atom% were within seven mass windows centered at the parent mass of this experimental spectrum. Each candidate peptide was scored based on the mass accuracy and goodness of fit between calculated b and y ions and measured b and y ions. Those candidate peptides with the highest score from the entire MS run were filtered using the target/decoy approach (Elias and Gygi, 2007) to achieve 1% FDR at the peptide level.

To set the ^15^N value above which a peptide would be declared “enriched”, multiple factors were considered. First, peptide ^15^N distributions from each sample's ^14^${\text{NH}}_{4}^{+}$ incubation were determined, and the value below which 99% of peptides fell set a 1% FDR for the declaration of enriched peptides. This value was 3% ^15^N in #3731 T_17d_, 4% in #3731 T_326d_, and 5% in #5133 T_160d_. Alternatively, we can consider the probability of unenriched peptides containing a range of ^15^N values using the binomial distribution. Using the mean enrichment value of the corresponding ^14^${\text{NH}}_{4}^{+}$ incubation as the baseline ^15^N frequency (0.45% for #3731 ^14^N T_17d_, 0.35% for #3731 ^14^N T_326d_, and 0.49% for #5133 ^14^N T_160d_), expected frequencies of X-length peptides containing Y% ^15^N can be calculated. When the probability of different ^15^N atom % values was plotted based on the binomial distribution and a best fit curve was calculated (*R*^2^ = 0.99924), the ^15^N enrichment value corresponding to a 1% likelihood of natural occurrence (i.e., the ^15^N value corresponding to a 99% confidence that it in fact was not enriched), was 0.1% for the shortest peptide (six amino acids). In a 53-residue peptide (the longest detected in this study), 70 N atoms are predicted; the likelihood of one ^15^N atom incorporation is much higher (24.6%) but the atom % ^15^N this represents is substantially lower (1.43%). There is a 4.2% chance of a 2.86% ^15^N enrichment, and a 0.47% chance of a 4.29% enrichment.

Of these statistical analyses, the most conservative 99% confidence interval surrounding an “enriched” designation is 5% ^15^N (derived from the ^15^N distribution in peptides recovered from the #5133 ^14^N sample). This value therefore demarcates enriched proteins in this study's analysis; the same enrichment level has been used as a threshold in other SIP studies (Pan et al., 2011; Justice et al., 2012).

### NanoSIMS analysis

Bulk protein extract from sample #3731 and cell aggregates from sample #5133 were analyzed with the Cameca nanoSIMS 50 L. Targets were mapped on the indium tin oxide-coated glass squares using both epifluorescence and transmitted light microscopy with a DeltaVision RT (Applied Precision Inc., WA) according to Dekas and Orphan (2011). For our analyses, a 2.5 pA cesium beam with a spot size of 100–200 nm was used. The beam rastered over areas dependent on the size of the aggregates selected (generally between 5 and 20 μm) with a 256 × 256 pixel resolution, a dwell time of 5–10 ms per pixel, and a resolving power of ~5000. ^15^N^12^C^−^ and ^14^N^12^C^−^ ions were collected for multiple frames, each lasting ~30 min. NanoSIMS images were processed with L'image (developed by L. Nittler, Carnegie Institution of Washington, Washington D.C.). Each set of frames was corrected for drift and detector dead time. *Clostridia* spores with known ^13^C/^12^C and ^15^N/^14^N ratios were used as a standard and measured before and after sample analysis to correct for instrumental mass fractionation and drift.

### Data availability

Sequence data can be found at the NCBI bioproject database, accession number PRJNA290197.

## Results and discussion

### Activity and community composition in seep sediment microcosms

Samples of methane seep sediment from Hydrate Ridge (hereafter referred to as #3731 and #5133) were each allocated into two anaerobic microcosm incubations overpressured with methane and amended with either ^14^${\text{NH}}_{4}^{+}$ (unlabeled treatment) or ^15^${\text{NH}}_{4}^{+}$ (labeled treatment; Figure 1). Cell aggregate abundance and sulfide concentrations, both of which correlate positively with sulfate-based AOM activity (Iversen and Jorgensen, 1985), were monitored throughout the incubation period. Both #3731 and #5133 were collected from actively seeping areas, yet the #3731 incubation, recovered from the lower 6–12 cm sediment horizon, demonstrated indications of lower metabolic activity (Table 2) and ^15^N incorporation (see below). Potential reasons for this discrepancy include differing *in situ* methane concentrations despite general markers of activity, localized heterogeneity of methanotrophic constituents (Table 2), and sediment horizon depth-related variability in AOM activity.

**Table 2:** **Data on incubation activity, as measured by sulfide production and cell abundance**.

**Days After Incubation Set-Up**		**#3731**					**#5133**			
	**#3731** ^**14**^**N**			**#3731** ^**15**^**N**		**#5133** ^**14**^**N**		**#5133** ^**15**^**N**	
	**Aggregate Counts (per mL)**		**Sulfide Concentration (mM)**	**Aggregate Counts (per mL)**	**Sulfide Concentration (mM)**	**Aggregate Counts (per mL)**	**Sulfide Concentration (mM)**	**Aggregate Counts (per mL)**	**Sulfide Concentration (mM)**
0	3.4 × 10^6^		0.9	3.4 × 10^6^	0.9	2.4 × 10^7^		2.4 × 10^7^	
6							1.2		1
17			1.4		2				
20							3.3		3.8
65			2.2		1.6		12.8		13
160						3.4 × 10^7^	14.3	4.1 × 10^7^	17.9
326	2.7 × 10^6^		4.2	7.9 × 10^5^	3				

Microbial communities were analyzed using 16S rDNA iTAG sequencing to compare the changes in diversity between the two time points of #3731 and between sediment #3731 and #5133 (Supplemental Data Sheet 3a). Across all three samples, the most abundant representatives were *Deltaproteobacteria* from the uncultivated SEEP-SRB1 (accounting for 7.2% of sequences on average, ± 1.3% SD) and SEEP-SRB2 (5.1 ± 1.7% SD) clades (Schreiber et al., 2010), *Sulfurovum* (6.7 ± 2.8% SD), and ANME-1a (6 ± 2.6% SD) and ANME-1b (5 ± 1.3% SD). ANME-2 subclades accounted for an average of 3.2% of sequences (±0.56% SD); however, the primer set used has been shown to preferentially detect ANME-1 over ANME-2 (Case et al., 2015), and independent FISH-based microscopy indicates a prevalence of ANME-2 consortia in the initial inoculum of both samples (data not shown). In all three samples, ANME sequences represented a substantial proportion of the archaeal relative abundance (67.8% on average, ± 6.3% SD). AOM-related enzymes of unspecified lineage putatively come from ANME organisms given that methanogenic lineages comprised < 0.1% of the archaeal community across all samples at the order level, as determined by 16S rRNA gene tag sequencing (Supplemental Data Sheet 3b).

### Protein identifications

The metaproteomic dataset produced here, using optimized extraction techniques and Orbitrap mass spectrometers, represents the deepest proteomic measurement of methane seep sediments to date and rivals most other environmental proteomics studies. Analyses at the 1% FDR level yielded 5664 unique proteins across all samples under the 1TP, 1UP condition, and 3495 unique proteins under the 2TP, 1UP condition (see Table 3 for quantification details). Compared with previous environmental studies (Supplemental Presentation 1; Table S1), the large number of proteins and selective incorporation of stable isotope label demonstrated here indicates that increased microbial diversity, low overall activity levels, and challenging physicochemical conditions are not insurmountable challenges in SIP-resolved metaproteomic studies. Nonetheless, the quantitative recovery, detection, and characterization of all proteins is not technically feasible because of protein adsorption to silicate minerals (Ding and Henrichs, 2002), complexation (Nguyen and Harvey, 2001) as well as the difficulty of solubilizing membrane-bound constituents (Tan et al., 2008).

**Table 3:** **The number of proteins identified in this study based on distinct identification criteria**.

**Sample**	**Protein Identifications**	
	**2TP 1UP**	**1TP 1UP**
Hydrate Ridge Seep Sediment #3731 ^14^N T_17d_	1562	2648
Hydrate Ridge Seep Sediment #3731 ^15^N T_17d_	1695	2741
Hydrate Ridge Seep Sediment #3731 ^14^N T_326d_	847	1477
Hydrate Ridge Seep Sediment #3731 ^15^N T_326d_	820	1484
Hydrate Ridge Seep Sediment #5133 ^14^N T_160d_	1850	3027
Hydrate Ridge Seep Sediment #5133 ^15^N T_160d_	1179	2260
Unique Proteins from All Samples	3495	5664

*2TP 1UP = two total peptides, one unique peptide required for positive identification; 1TP 1UP = one total peptide, one unique peptide required for positive identification*.

A core set of 456 (1TP, 1UP) and 283 (2TP, 1UP) proteins were detected in all six samples; of these, the five most common categories were “proteins of unknown function” with best matches to hypothetical genes (comprising 31.1% of protein identifications averaged between the search conditions), adenylyl sulfate reductase subunit A (AprA, 7.4%), McrB (5.7%), McrG (4.9%), and ATPase subunit A (4.6%). Phylogenetic assignments of the cumulative metaproteome demonstrated a higher relative abundance of Bacteria-classified proteins (56.9% 1TP, 1UP; 58.7% 2TP, 1UP) than those assigned to Archaea (37.2% 1TP, 1UP; 35.2% 2TP, 1UP).

### ^15^N enrichment of proteins expressed in AOM sediment incubations

To provide additional context for ^15^N incorporation-based activity levels, the isotopic composition of bulk protein extract and intact ANME-SRB consortia was measured by nanoscale Secondary Ion Mass Spectrometry (nanoSIMS). NanoSIMS analysis of #3731 sediment bulk protein extracts from the ^15^${\text{NH}}_{4}^{+}$ amended incubation revealed a slow-growing community with a low but measurable enrichment at the early (0.45 atom % enrichment for ^15^N T_17*d*_) and late (0.53 atom % at ^15^N T_326*d*_) time points compared to the ^14^${\text{NH}}_{4}^{+}$ treated controls (0.38 atom % at both time points; Table 4). *In silico* identification of enriched peptides from sample #3731 also exhibited a small but detectable isotopic enrichment of the protein pool during the 326-day incubation. The ^15^N atom % enrichment of the entire #3731 ^15^N peptide pool based on Sipros analysis was 0.4% at T_17*d*_, increasing to 0.8% at T_326*d*_ (while corresponding values for ^14^N control incubations were 0.5 and 0.4%, respectively).

**Table 4:** **^**15**^N enrichment values of proteome-derived peptides and bulk protein extract**.

**Sample ID**	**Incubation Time (d)**	**Peptide LC MS/MS Analysis (SE)**		**Bulk Protein Extract nanoSIMS Analysis**
		**^15^N Atom % Mean**	**% of Enriched Peptides (>5% ^15^N)**	**^15^N Atom % Mean (SE)**
#3731 ^14^N T_17d_	17	0.5 (0.03)	0.44 (0.03)	0.38 (0)
#3731 ^15^N T_17d_	17	0.4 (0.01)	0.52 (0.07)	0.45 (5x10-5)
#3731 ^14^N T_326d_	326	0.4 (0.09)	0.62 (0.07)	0.38 (0)
#3731 ^15^N T_326d_	326	0.8 (0.04)	1.48 (0.04)	0.53 (5x10-5)
#5133 ^14^N T_160d_	160	0.5 (< 0.01)	0.87 (0.03)	NA
#5133 ^15^N T_160d_	160	9.5 (18.34)	18.06 (0.68)	NA

Peptides recovered from sample #5133 ^15^N after 160 days of incubation with ^15^${\text{NH}}_{4}^{+}$ demonstrated significantly more uptake of the ^15^N label, with Sipros-calculated isotopic enrichment of 9.5 atom % ^15^N, while peptides from the corresponding ^14^${\text{NH}}_{4}^{+}$ control exhibited a mean enrichment of 0.5%. NanoSIMS analysis of ANME-SRB consortia from the same sample showed substantially higher ^15^N incorporation levels, reaching 23.8% after 64 days (Supplemental Presentation 1; Figure S1). The broader peptide-based enrichment value may provide a more representative constraint on community-wide anabolic rates because ANME-SRB consortia constitute a particularly active subset of the community (Knittel and Boetius, 2009). Peptides that uniquely mapped to ANME archaea had a mean ^15^N value of 13.8%, while those attributed to the dominant deltaproteobacterial sulfate-reducing group (*Desulfobacterales*) had a mean enrichment value of 10.1%. Proteins previously linked to the reverse methanogenesis pathway had a mean enrichment of 18.3%, whereas proteins associated with dissimilatory sulfate reduction exhibited a mean enrichment of 9.3%. These values suggest that ANME lineages responded to the methane incubation conditions with heightened biosynthetic activity compared to SRB, and that a higher proportion of reverse methanogenesis enzymes were newly synthesized compared to other identified ANME proteins.

The observation of enhanced ^15^N incorporation in the nanoSIMS analysis of individual intact AOM consortia relative to the calculated ^15^N values from bulk ANME and SRB-associated proteins recovered from the same incubation is not fully understood. Potential explanations include the incomplete detection of ANME and deltaproteobacterial proteins, enrichment discrepancies among organisms or different cellular nitrogen sources, or variation in the turnover of proteins and other cellular nitrogen stores (Reitzer, 2003). Membrane proteins are believed to comprise 20–30% of prokaryotic proteomes based on genomic analysis (Stevens and Arkin, 2000), yet solubilizing membranes and accessing these proteins is a technical challenge (Trötschel and Poetsch, 2015); preferential incorporation of ^15^N into membrane proteins, such as receptors, transporters, or oxidoreductases may be partially responsible for the ^15^N unaccounted for in our SIP metaproteomic analysis.

**Figure 2:**
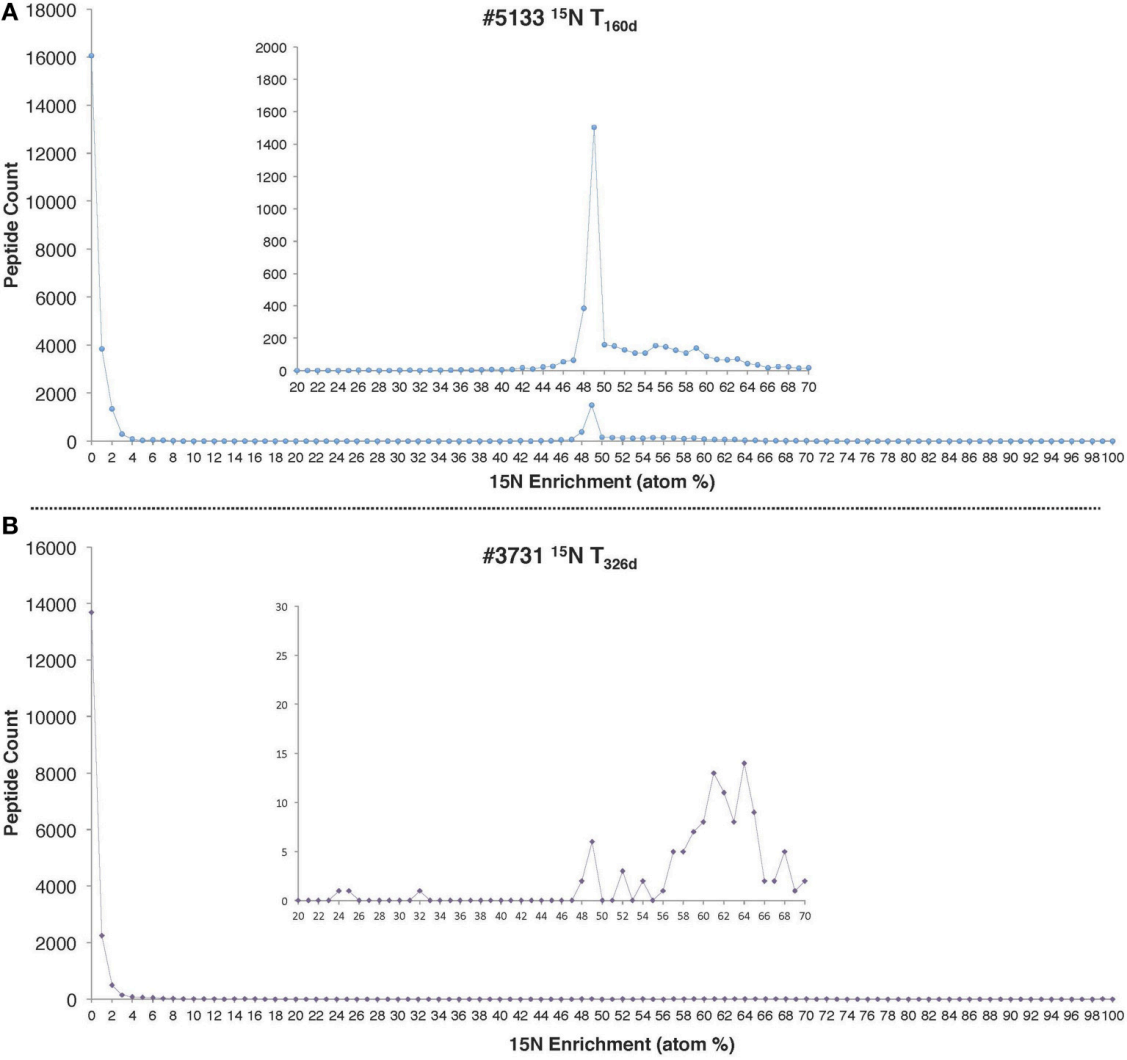
**^**15**^N enrichment distributions for all peptides identified in (A) #5133 ^**15**^N T_**160**d****_ and (B) #3731 ^**15**^N T_**326**d****_ incubations**. In both cases, the precursor pool of ${\text{NH}}_{4}^{+}$ was 496 μM and 1 mM of ^15^${\text{NH}}_{4}^{+}$ was added at the beginning of the incubation period.

Peptide enrichment values exhibited a marked bimodal distribution, with an unenriched peak and a ^15^N enriched peak at 49% for the #5133 ^15^N T_160d_ sample and 64% for the #3731 ^15^N T_326*d*_ sample (Figure 2). The presence of two distinct peptide pools in the data presented here implies that most of the detected proteins (made up of peptides with ^15^N values below 5%) were likely synthesized before the addition of ^15^${\text{NH}}_{4}^{+}$ and were not fully degraded, while the remainder was newly synthesized. The lack of peptides with intermediate enrichment values argues against multiple protein precursor pools (Justice et al., 2014) or substantial heterotrophic turnover of the system's primary producers during the course of the 160-day and 326-day incubations.

### Functional and phylogenetic distributions of enriched proteins

Of the proteins identified from the #5133 ^15^N sample, 32% (*n* = 377) were isotopically enriched using the conservative (2TP, 1UP) criterion. To determine which functional traits or phylogenetic affiliations may be disproportionately represented in either the enriched or unenriched protein pools, annotated proteins were partitioned by enzyme type or phylogenetic assignment of the corresponding ORF. The relative abundance of these categories was calculated for both the enriched and unenriched proteins, and the variance contributed by each bin was determined (calculated by the square of the difference in relative abundances).

**Figure 3:**
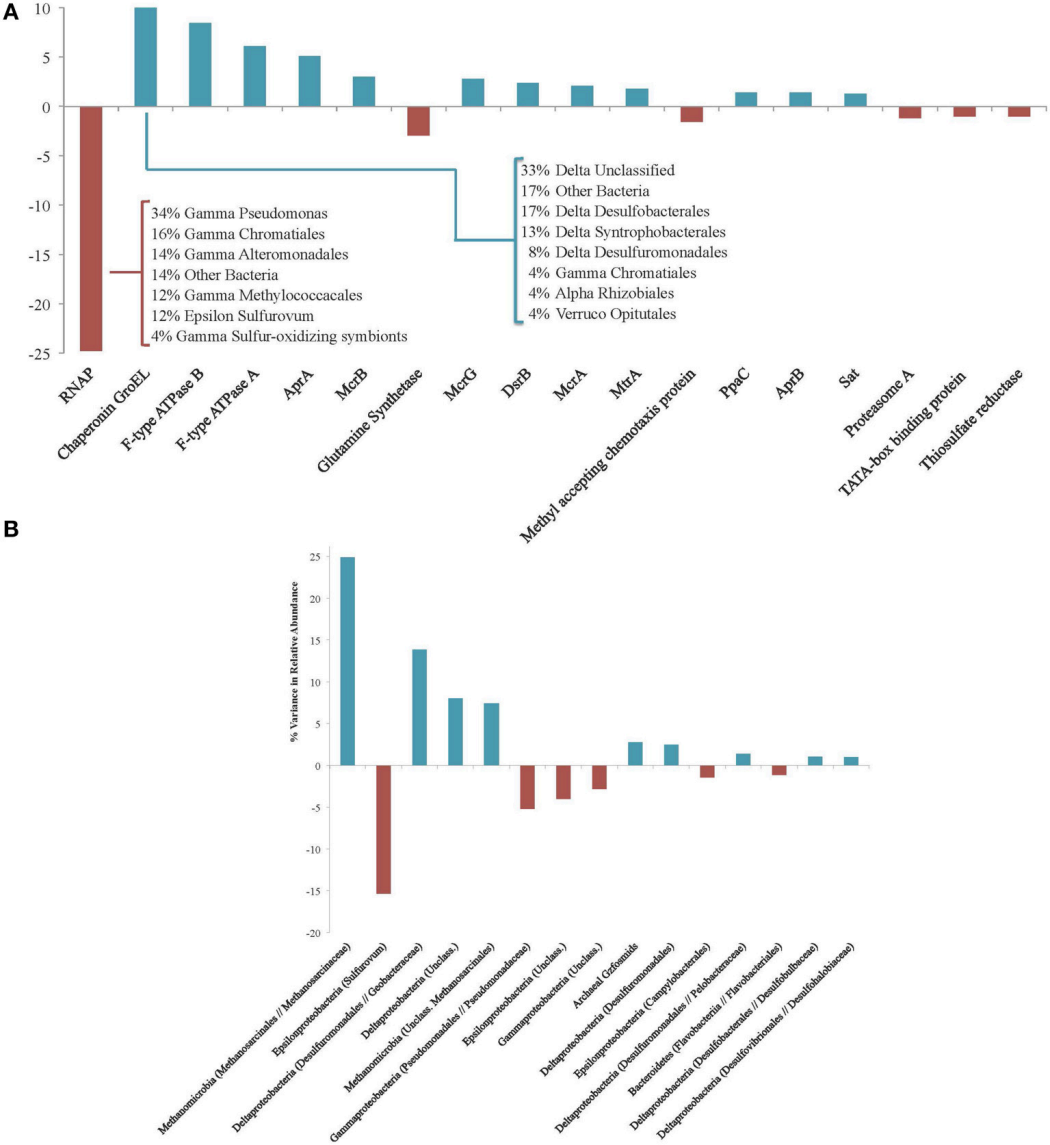
**(A)** Protein functional groups accounting for at least 1% of variance between the enriched and unenriched #5133 ^15^N T_160d_ protein pools. Positive values reveal protein types that are more abundant in the enriched fraction; negative values indicate those more prevalent in the unenriched fraction. Inset table shows the phylogenetic affiliations of unenriched RNAP and enriched GroEL proteins. **(B)** Phylogenetic associations accounting for at least 1% of variance between the enriched and unenriched #5133 ^15^N T_160d_ protein pools. Positive values reveal phylogenetic assignments whose protein products are more abundant in the enriched fraction; negative values indicate those more prevalent in the unenriched fraction. Phylogenetic assignments were made at the family level; higher level assignments are provided if no family-level specificity was available, and genus-level assignments are provided if no other genera were observed in the same family. Archaeal Gzfosmids are ANME-affiliated sequences reported by Hallam et al. (2004).

Eighteen functional protein categories accounted for 79.8% of the variance between enriched and unenriched proteins, with each contributing at least 1% of the variance (Figure 3A). The largest contributor, whose distribution produced 24.8% of the observed variance, was RNA polymerase (RNAP), which was more prominent in the unenriched protein pool. By examining the phylogenetic assignments of RNAP orthologs, the enzyme serves as a representative signal of organism-wide regulation. The detection of remnant, unenriched RNAP proteins reveals their resistance to degradation over the course of the 160-day experiment and offers the possibility that all transcription needs were met by pre-existing, long-lived copies. The relative lack of newly produced copies could indicate that the incubation conditions were deleterious to active transcription and, perhaps, survival of some microorganisms; this scenario is consistent with the detection of few enriched proteins from similar phylogenetic groupings, suggesting a broader shutdown of transcriptional machinery. In this context, the lineages responding most negatively to the anaerobic incubation conditions include *Methylococcales, Sulfurovum, Chromatiales, Alteromonadales*, and *Pseudomonas* (Figure 3A), indicating a shift away from putative oxygen- or nitrate-based metabolisms common within the oxidizing conditions of upper seep sediment layers.

Of the predominantly enriched protein types in #5133, chaperonin GroEL accounts for the most variance (10.9%) between the enriched and unenriched pools, followed by F-type ATPase subunits B (8.4%), and A (6.1%). GroEL is frequently detected in environmental metaproteomic studies (Benndorf et al., 2007; Sowell et al., 2009; Verberkmoes et al., 2009) and has been demonstrated to prevent amorphous protein aggregations (Hartl et al., 2011), facilitate proper folding of a wide variety of enzymes (Kerner et al., 2005), and mediate stress response (Kim et al., 2013); changing pressure or energetic regimes in our incubations may have prompted such a response. F-type ATPase and pyrophosphatase PpaC (1.4%) are both involved in ATP generation, concordant with previous observations of heightened energy production under conditions of physiological stress (Alexandre et al., 2001) such as those potentially experienced by slow-growing methanotrophic consortia (Valentine, 2007). Several high-variance proteins are involved in reverse methanogenesis and sulfate reduction pathways. AprA (5.1%), McrB (3.0%), McrG (2.8%), sulfite reductase subunit B (DsrB; 2.4%), McrA (2.1%), MtrA (1.7%), AprB (1.4%), and sulfate adenylyltransferase (Sat, 1.3%) were all more prevalent in the ^15^N enriched fraction. This observation is consistent with active sulfate-coupled AOM as demonstrated by sulfide production during the incubation as well as quantitative increases in and growth of ANME-SRB consortia (Table 2, Supplemental Presentation 1, Figure S1).

Of the high-variance proteins that were detected at higher levels within the unenriched fraction, methyl-accepting chemotaxis proteins (accounting for 1.6% of the variance) may be used in biofilm-linked cell signaling or orientation toward favorable environmental conditions (Morgan et al., 2006). Most of the observed chemotaxis proteins exhibit greatest homology with *Gammaproteobacteria*, and their near-absence among ^15^N-enriched proteins suggests decreased need for mobility-linked sensing and/or less communication among these constituents. Glutamine synthetase (3.0%) catalyzes the formation of glutamine from glutamate and ammonia, and its minimal presence among enriched proteins could indicate the accessibility of free glutamine (and likely other amino acids) from biomass degraded during the incubation, or a heightened reaction rate—facilitated by increased ${\text{NH}}_{4}^{+}$ concentrations—that obviated new enzyme production. Predominantly unenriched proteins may reveal functions of lower importance under incubation conditions, while reflecting remnant capabilities that highlight the importance of proteins' ability to outlive upstream markers of activity (e.g., transcripts (Moran et al., 2013).

Fifteen phylogenetic bins each contributed at least 1% of the variance between the relative abundances of enriched and unenriched proteins recovered from #5133 ^15^N, representing 92.5% of the total variance (Figure 3B). Among the more notable differences was the prevalence of enriched proteins linked to methane-metabolizing lineages (accounting for 32.3% of the variance) and *Deltaproteobacteria* (27.7%), particularly orders with known sulfate reducers (18.3%) expressing proteins from carbon fixation and sulfate reduction pathways, GroEL, and ribosomal proteins. Proteins attributed to *Epsilonproteobacteria* (20.8%) and especially *Sulfurovum* RNAP, Apr, and ATPase (15.3%) were markedly less prevalent in the enriched pool. The dearth of enriched epsilonproteobacterial protein products is consistent with these organisms' proposed susceptibility to changing conditions (Alain et al., 2004; Sievert et al., 2007; Toner et al., 2012) as well the potentially unfavorable conditions for supporting active oxygen- or nitrate-coupled sulfide oxidation. The high relative abundance of *Sulfurovum* in the #5133 tag sequencing data (Supplemental Data Sheet 3a) highlights the utility of SIP proteomics: the abundance and persistence of 16S rRNA genes and proteins may not always be reflective of biosynthetically active participants in an environment.

Finally, of the proteins that remained unannotated (i.e., proteins of unknown function using the 2TP, 1UP criterion), 26 were enriched in the #5133 ^15^N T_160d_ sample and were present in all six proteomes, suggesting they are important in seep sediment habitats and are actively synthesized under conditions supporting AOM. Approximately half of the unknown proteins were attributed to uncultured *Archaea* while most of the remainder was associated with sulfate-reducing *Deltaproteobacteria* (Table 5). Up to 40% of expressed bacterial proteins currently lack functional classification, and there is a growing need for further categorization and characterization of these proteins (Galperin and Koonin, 2004). These 26 consistently detected, uncharacterized proteins represent notable targets for functional biochemical study (Guengerich and Cheng, 2011; Goodacre et al., 2014).

**Table 5:** **UniProt results for the 26 proteins that were detected in all six proteomes, enriched in #5133 ^**15**^N T_**160**d****_, and were unannotated via KAAS and UniProt**.

	**UniProt Annotation**	**Phylogenetic Assignment**	**UniProt ID**	**% Identity**	***E-*value**
3datasets_contig_19395_3	Putative uncharacterized protein	Uncultured archaeon	D1J9F4^**^	51.7	2 E-64
5datasets_contig_137335_2	Putative uncharacterized protein	Uncultured archaeon	D1J9F4^**^	90.5	1 E-45
5datasets_contig_214909_1	Putative uncharacterized protein	Uncultured archaeon	D1J9F4^**^	79.1	2 E-47
5datasets_contig_354507_3	Putative uncharacterized protein	Uncultured archaeon	D1J9F4^**^	88.6	1 E-38
5datasets_contig_426791_2	Putative uncharacterized protein	Uncultured archaeon	D1J9F4^**^	61.2	2 E-64
VO1_contig_3180_1	Putative uncharacterized protein	Uncultured archaeon	D1JGR9^**^	78.5	4 E-42
3datasets_contig_52223_1	Hypothetical secreted protein^*^	Uncultured archaeon	D1JHB5^**^	42.9	2 E-61
3datasets_contig_128337_2	Hypothetical secreted protein^*^	Uncultured archaeon	D1JHB5^**^	38.1	3 E-52
5datasets_contig_120634_2	Hypothetical secreted protein^*^	Uncultured archaeon	D1JHB5^**^	46.6	3 E-55
3730_contig_6723_4	Putative uncharacterized protein	Uncultured archaeon	D1JHC5^**^	77	5 E-35
5datasets_contig_264150_1	Putative uncharacterized protein	Uncultured archaeon	Q2Y4L6^**^	57.5	3 E-62
3730_contig_4042_9	Uncharacterized protein	Uncultured archaeon	Q6MZD7^**^	52.2	4 E-44
3datasets_contig_80833_1	Uncharacterized protein	Uncultured archaeon	Q6MZD7^**^	54.1	4 E-42
VO1_contig_16363_1	Uncharacterized protein	Uncultured archaeon	Q6MZD7^**^	52.6	2 E-41
5datasets_contig_14875_4	Uncharacterized protein	Desulfococcus oleovorans	A8ZRQ4	36.1	5 E-67
3730_contig_10399_1	Uncharacterized protein	Desulfonatronospira thiodismutans	D6SNE1	62.3	6 E-42
3730_contig_109916_2	Uncharacterized protein	Desulfonatronospira thiodismutans	D6SNE1	63.8	4 E-44
5datasets_contig_13906_2	Putative uncharacterized protein	Uncultured Desulfobacterium sp.	E1YFV9	62.6	0 E+00
5datasets_contig_110039_2	Putative uncharacterized protein	Desulfobulbus propionicus	E8RH53	62.7	5 E-49
3730_contig_26432_2	Hypothetical protein	Desulfatirhabdium butyrativorans	UPI0003FB5DCE	45.7	5 E-101
5datasets_contig_120508_2	Hypothetical protein	Desulfobulbus japonicus	UPI0003FCAF71	54.9	2 E-32
5datasets_contig_26951_1	Hypothetical protein	Desulfosarcina sp. BuS5	UPI0004837EE9	55.8	0 E+00
5datasets_contig_175666_3	Hypothetical protein	Thermodesulfatator atlanticus	UPI0003B3E199	40.4	2 E-39
5datasets_contig_93287_2	Putative uncharacterized protein	Moorea producens	F4XS28	47.4	7 E-131
5datasets_contig_80150_1	Hypothetical protein	Longispora albida	UPI000381E695	43.8	5 E-105
5datasets_contig_244181_2	Uncharacterized protein^*^	Uncultured bacterium	K1YAD4	51.4	1 E-114

* *Contains an InterPro-predicted transmembrante domain*.

** *Best-match ORFs derived from Meyerdierks et al. (2010)*.

### Metabolic pathways

#### Methane metabolism

**Figure 4:**
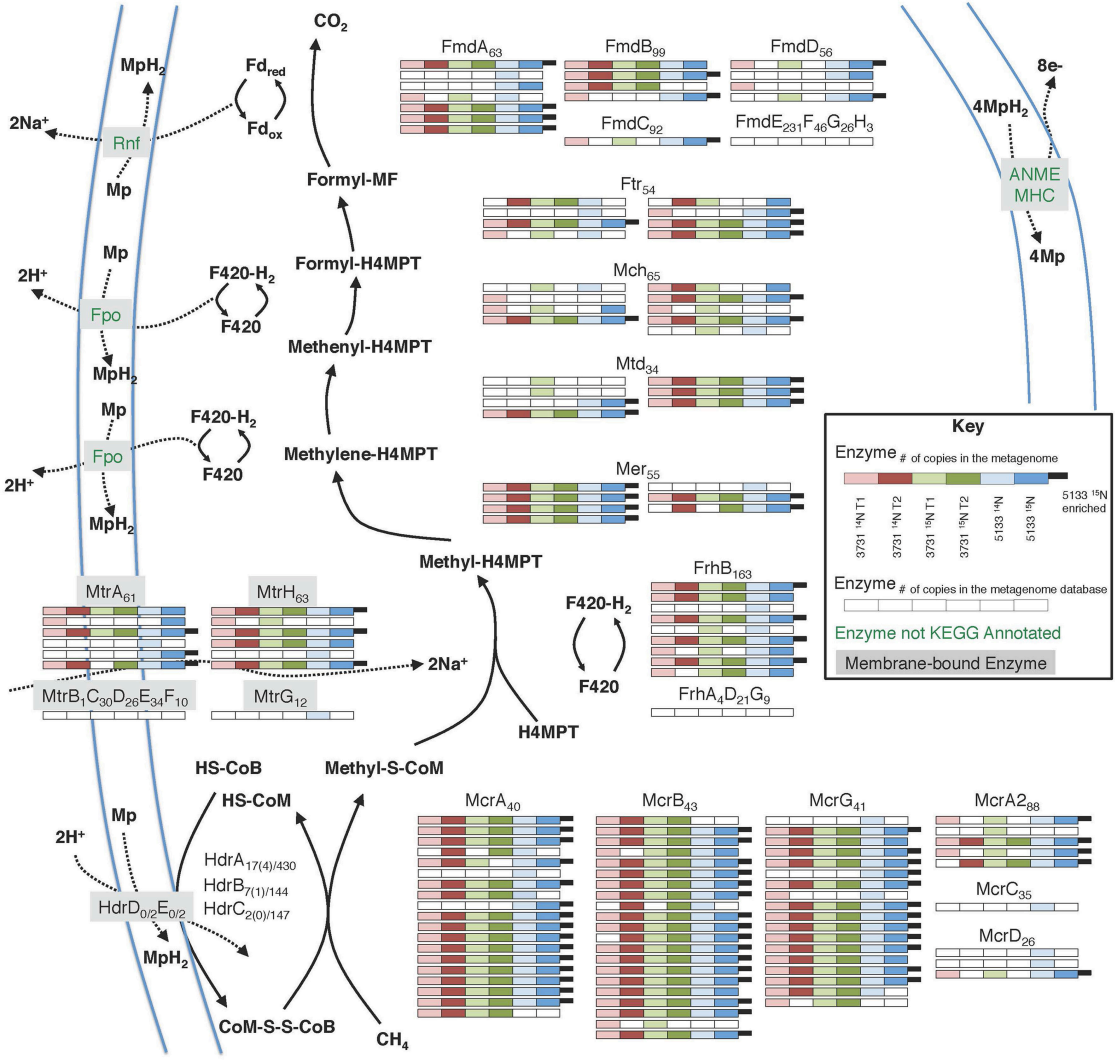
**Metaproteomic data for enzymes involved in the reverse methanogenesis pathway**. Filled boxes indicate an enzyme constituent's presence in the associated sample's proteome. Due to space constraints, Hdr detections are not shown, but relevant information is provided as Protein _orthologsdetected(enrichedorthologs)∕orthologsinmetagenomicdatabase._

The anaerobic oxidation of methane (AOM) is a dominant metabolism at Hydrate Ridge methane seeps. Many distinct orthologs of all enzymes involved in the reverse methanogenesis pathway were recovered from incubated seep sediment (Figure 4; phylogenetic affiliations provided in Supplemental Data Sheet 4a), signifying unprecedented completeness and diversity of pathway protein detection. Methyl-coenzyme M reductase (Mcr) is one of the most abundant enzymes in ANME, accounting for 7% of total extractable proteins from the ANME-1 rich Black Sea mats (Krüger et al., 2003). Across all six of our proteomic data sets, 10.4% of peptides were attributable to Mcr; 34.6% of ^15^N enriched peptides in sample #5133 were associated with this critical enzyme. Considerable diversity of detected Mcr proteins was observed, with approximately 20 orthologs of each Mcr subunit identified across all six samples, representing 47% of the mcr protein sequences within our seep metagenome database. The #5133 microcosm thus appeared to stably maintain a genetically diverse population of active ANME archaea over the course of the extended incubation. Of the expressed alpha, beta, and gamma subunits of MCR, one-third was affiliated with ANME-1 (31% of detected orthologs, see Supplemental Data Sheet 4a), with smaller contribution of ANME-2c (19%), Methanosarcinaceae (14%), and ANME-2a derived (12%) orthologs. The three non-catalytic Mcr components (McrA2, McrC, McrD) were also identified, but these putative (methanogenic) activation domains (Prakash et al., 2014) were notably less prevalent in the metaproteome. This result may indicate McrA2, McrC, and McrD re-use as activating components, or their decreased relevance under methanotrophic conditions, potentially reflective of a methane-oxidizing enzymatic mechanism that is distinct from a strict reversal of methane formation.

Several previous ANME-targeted meta-omics efforts have sought to reconstruct the reverse methanogenesis pathway (Table 6; e.g., Hallam et al., 2004; Meyerdierks et al., 2010; Stokke et al., 2012; Haroon et al., 2013; Wang et al., 2014), but the Mer protein in particular has proven challenging to identify (Hallam et al., 2004; Meyerdierks et al., 2010; Stokke et al., 2012). In our proteomic experiments, seven Mer orthologs were identified (six of which were ^15^N-enriched), exhibiting best homology to ANME-2a, *M. burtonii*, and other *Methanosarcinales* lineages. Our detection of ^15^N-enriched Mer orthologs under incubation conditions favoring AOM is consistent with a reverse methanogenesis metabolism. It is possible that ANME-1 methane oxidation proceeds via the *mer* bypass (Meyerdierks et al., 2010), whereby activated methane is converted first to methyl-containing intermediates and then to formaldehyde, which is processed to methylene-H_4_MPT by a formaldehyde-activating enzyme (Fae) and hexulose-6-phosphate (Hps). The viability of the methanogenic version of this pathway has been demonstrated with an *M. barkeri mer* deletion mutant (Welander and Metcalf, 2008). An ANME-1 *mer* copy has not yet been identified, and 12 orthologs of the Fae/Hps fusion protein were detected across all samples (1TP, 1UP).

**Table 6:**
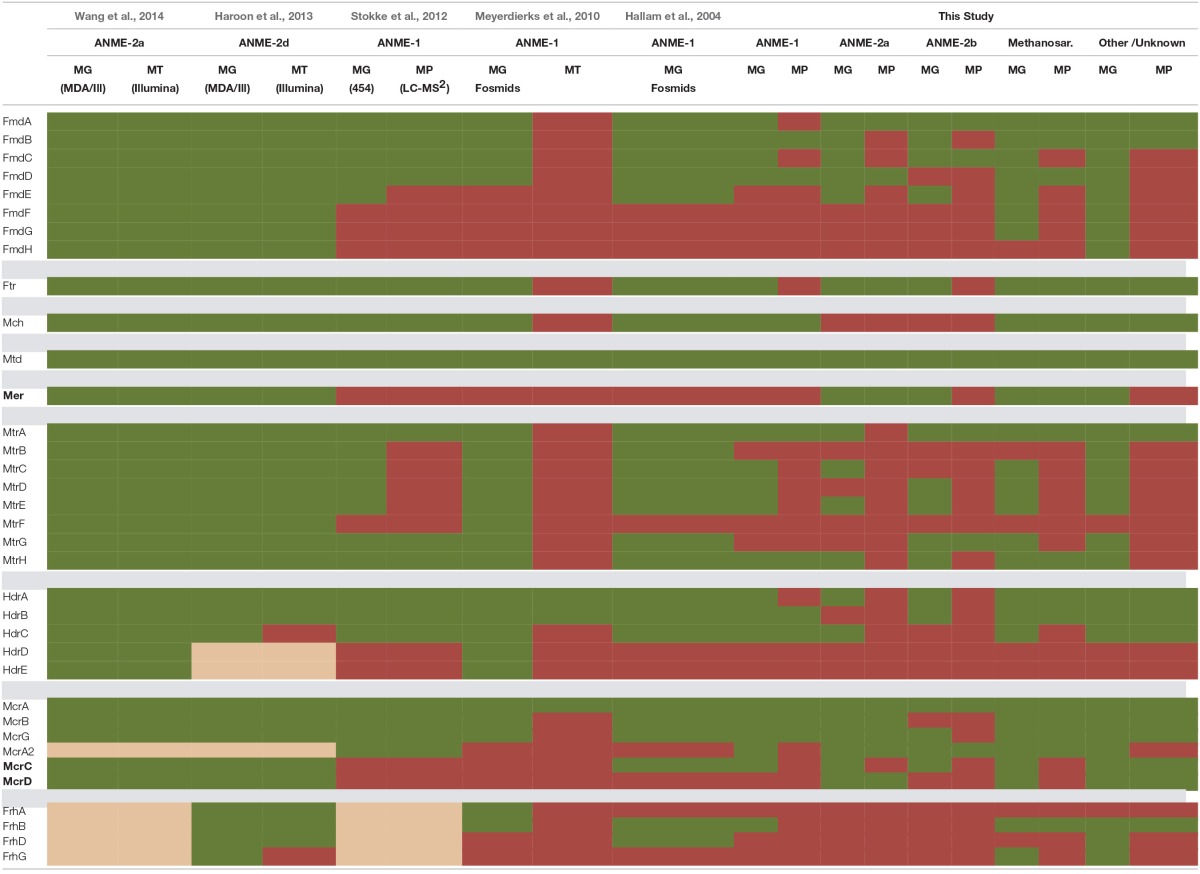
**Central reverse methanogenesis enzymes identified in metagenomic, metatranscriptomic, and metaproteomic analyses**.

Green, identified; orange, not addressed; red, not identified. ANME proteins newly detected in seep environments during this study are highlighted in bold text. (McrABG subunits associated with ANME-2c derived fosmids were also detected; no other ANME-2c genes related to reverse methanogenesis were in the metagenomic database.)

Building upon the 356 proteins detected from seep sediments in the Nyegga area (Norwegian Sea; Stokke et al., 2012), additional subunits of enzymes putatively involved in the reverse methanogenesis pathway were newly detected in our study, including McrC and McrD (Table 6). Of the components not found in the proteome, some of those present in the co-located metagenomes (MtrBCDEF, HdrDE) are membrane-bound proteins (Thauer et al., 2008) and thus are expected to be more difficult to quantitatively recover (Trötschel and Poetsch, 2015). Given the relatively poor genomic coverage of the ANME-2 lineages, combined with the observed dominance of ANME sequences relative to methanogens in our iTAG diversity surveys (Supplemental Data Sheet 3b), it is likely that most of the reverse methanogenesis pathway orthologs of poor homology derive from ANME representatives.

#### Mcra post translational modifications

**Figure 5:**
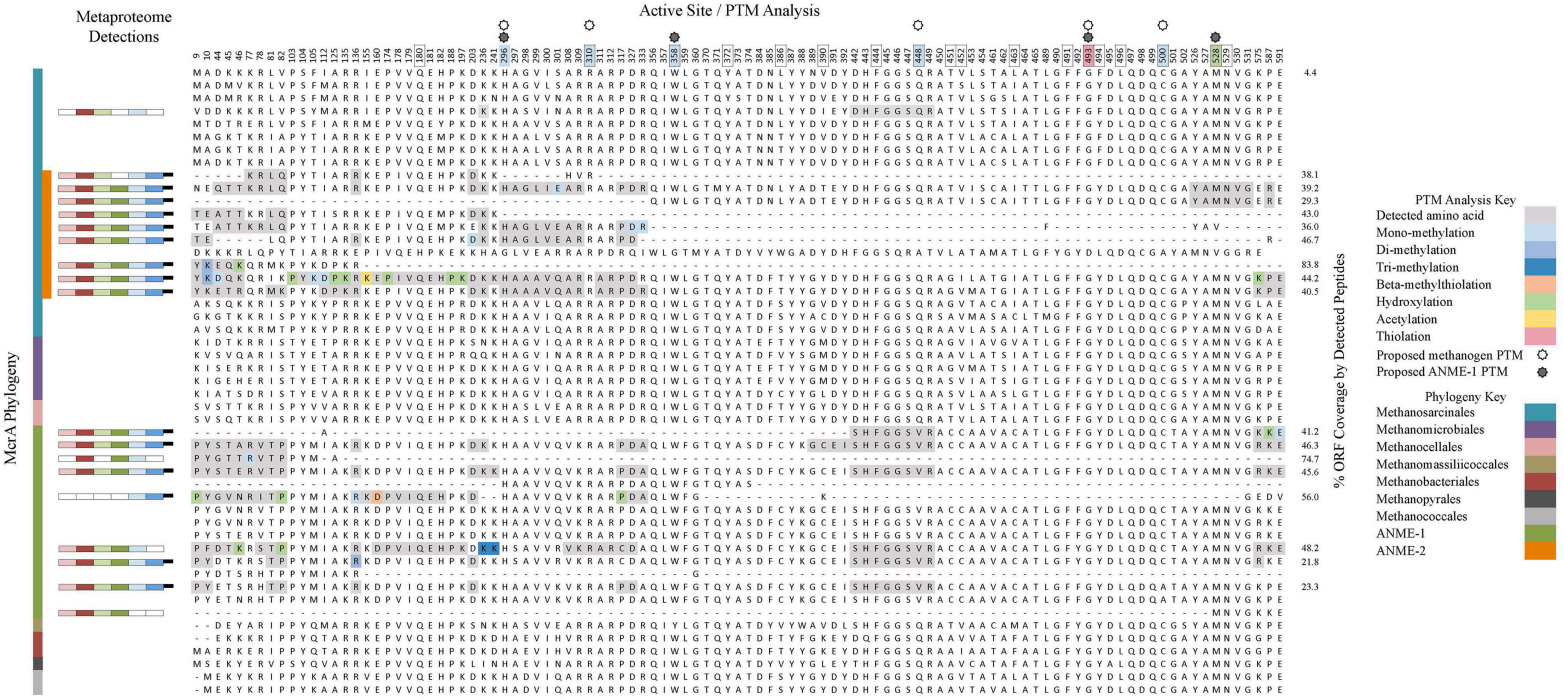
**A phylogenetic and PTM-based analysis of the McrA subunit**. The left panel shows a Muscle-aligned phylogenetic tree of all McrA sequences in the metagenomic database, as well as additional cultured organisms to account for all seven methanogenic orders and other ANME representatives. The metaproteomic detection panel shows the presence/absence bars from Figure 4, positioned next to their corresponding ORF label. The active site/PTM analysis panel provides alignments generated by Jalview for selected amino acid positions of note that together account for all PTMs observed in this and previous studies, as well as residues implicated in the enzyme's active site. Amino acids detected in proteomics analyses are highlighted in gray; colored residues exhibited PTMs specified in the figure key. PTMs observed in previous studies (Elias and Gygi, 2007; Sharma et al., 2012; Wang et al., 2013) are portrayed by color-coding of amino acid position numbers at the top of the alignment; boxed numbers correspond with active site residues described in Sharma et al. (2012). ORF coverage was calculated by dividing the number of amino acids in an ORF's detected peptides by that ORF's full length. All PTM analysis only includes data from #5133 ^14^N T_160d_.

Mcr is a critical enzyme in the reverse methanogenesis pathway, activating methane with a disulfide bond and initiating the multi-step anaerobic oxidation of methane (Krüger et al., 2003; Shima and Thauer, 2005). To better understand the range of variability found between McrA orthologs and potentially distinguish between methanogenic and methanotrophic forms of the enzyme, we conducted a computational search for selected post-translational modifications (PTMs; Camacho et al., 2009). MS-based PTM analysis is a developing field, and it is difficult to draw functional conclusions from detected modifications; nonetheless, the approach has been used to corroborate modifications (Selmer et al., 2000) and as a discovery tool supplemented by subsequent crystallographic confirmation (Moellering and Cravatt, 2013). Spectra from sample #5133 ^14^N T_160d_ were used due to its enhanced metabolic activity relative to #3731 (Table 2) and unlabeled peptides, which ensured the accuracy of PTM mass windows. PTMs were observed among 10 of the 18 McrA orthologs, 13 of 19 McrB orthologs, and 7 of 17 McrG orthologs; none were identified on five McrA2, one McrC, and three McrD orthologs. Twenty-eight distinct modification events of McrA were observed, including mono-, di-, and tri-methylations, hydroxylation, beta-methylthiolation, and acetylation (Figure 5). PTMs were detected with high frequency (occurring an average of once every 84 amino acids among ANME Mcr subunits), but only 11% were affiliated with multiple orthologs. This observation, in addition to their positioning away from active site residues, suggests a dominance of PTMs responsive to environmental variation (Olsen et al., 2010) that may be involved in enzyme regulation (Olsen and Mann, 2013).

The frequent occurrence and differential expression of PTMs among McrA orthologs is a discovery that suggests modifications are common and are phylogenetically and/or environmentally heterogeneous. Functional traits can be substantially affected by the expression of such modifications (Moellering and Cravatt, 2013) and they represent potentially fruitful targets for future work aimed at distinguishing enzymatic modulation or differentiating methanogenic from methanotrophic Mcr *in vivo*.

#### Sulfur metabolism

**Figure 6:**
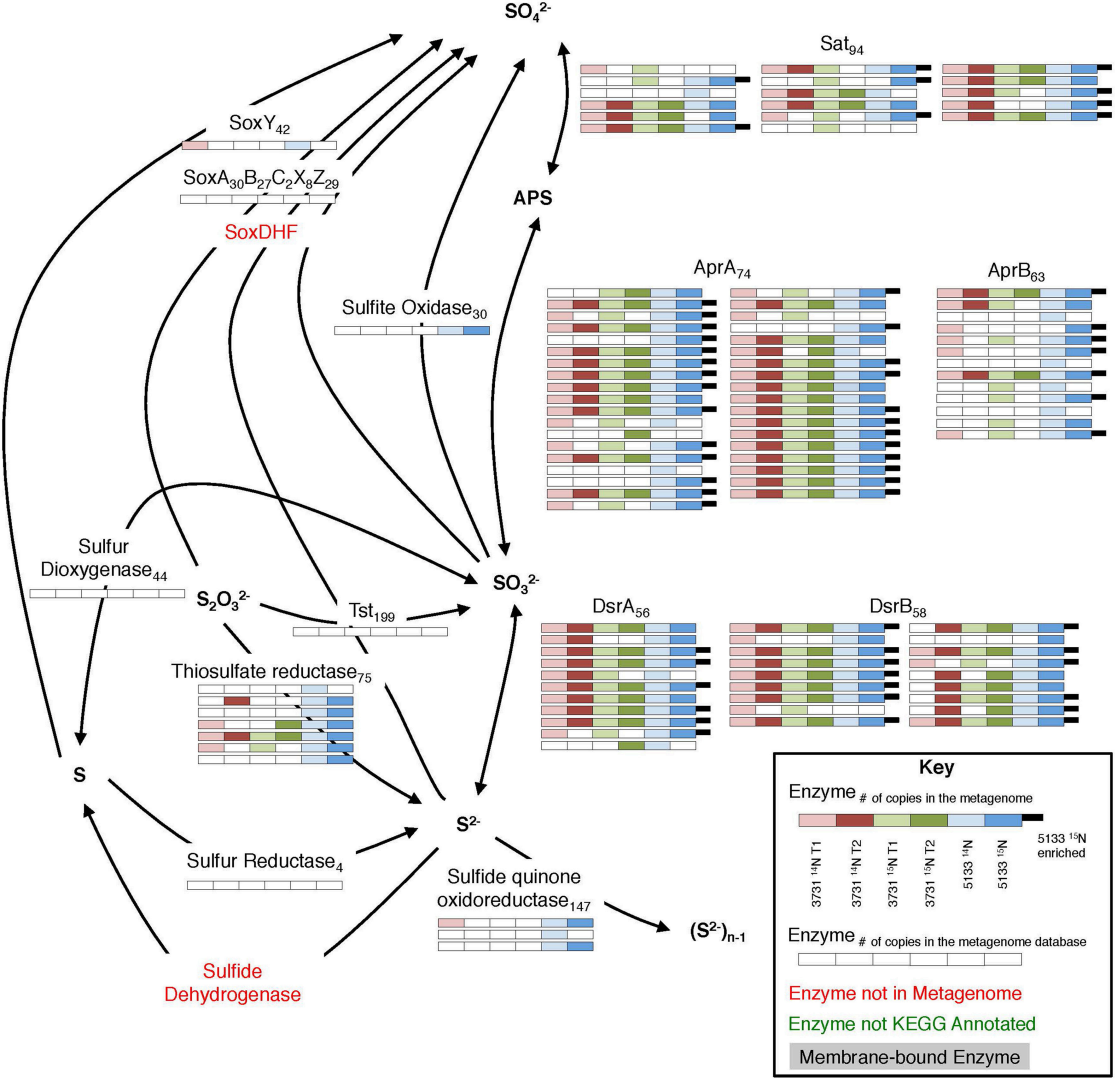
**Metaproteomic data for enzymes involved in the sulfate reduction pathway**. For key, see Figure 4.

The dissimilatory sulfate reduction pathway was well-represented in the metaproteomes from all six seep sediment incubation samples (Figure 6). In total, 17 Sat, 37 AprA, 13 AprB, 11 DsrA, and 18 DsrB proteins were recovered, accounting for 28% of the potential metagenomic search space among these enzyme subunits. Previous protein-based examinations of sulfate reduction in methane seep environments have reported a complete sulfate reduction pathway within the ANME-1 dominated “reefs” in the Black Sea (Basen et al., 2011) and have localized AprB (Wrede et al., 2012), Sat, and Dsr enzymes (Milucka et al., 2013) to ANME-affiliated sulfate SRB via antibody detection. Sulfate reduction pathway proteins were also recovered from Nyegga seep sediments, with the exception of DsrB (Stokke et al., 2012).

Phylogenetic affiliations of sulfate reduction pathway orthologs (Sat, AprA, AprB, DsrA, and DsrB) identified across all six proteomic experiments are shown in Supplemental Data Sheet 4b,c. Of those classified to class level, deltaproteobacterial enzymes (84%) dominated, with *Desulfobacterales* as the most common family (39% of all class-level partitioned orthologs). Based on the 16S rDNA community data, the majority of the deltaproteobacteria were members of the SEEP-SRB1 and SEEP-SRB2 clades within the *Desulfobacterales* (Supplemental Data Sheet 3a), an observation supported by FISH visualization (Trembath-Reichert et al., 2016) and the frequent SEEP-SRB association with ANME (Orphan et al., 2002; Knittel et al., 2005).

Sulfate-coupled oxidation of non-methane hydrocarbons can also play a substantial role in sulfur metabolic processing at hydrocarbon seeps (Bose et al., 2013). This process is typically attributed to the DSS clade, with additional contributions from non-DSS *Desulfobacteraceae, Desulfobulbaceae, Syntrophobacteraceae*, and *Desulfurellales* (Widdel et al., 2010; Kleindienst et al., 2014). It is difficult to attribute the presence of these organisms exclusively to higher-order alkane oxidation, but Sat enzymes linked to *Syntrophobacteraceae* were detected, and all of the above lineages were represented in the 16S rRNA gene tag data (Supplemental Data Sheet 3a). 90 (1TP, 1UP) proteins—89 of which did not correspond with previously annotated proteins—demonstrated closest homology to the *Desulfosarcina* strain BuS5, the only pure culture SRB known to degrade short chain alkanes (Kniemeyer et al., 2007).

Seven thiosulfate reductase orthologs attributed to *Delta*- and *Epsilonproteobacteria* were detected (2TP, 1UP), suggesting that thiosulfate reduction is a relatively common metabolic capability within seep sediments. This observation is consistent with previous evidence of thiosulfate reduction from ANME-1-rich seep sediment incubations from Eckernförde Bay (Jagersma et al., 2012) and thiosulfate disproportionation in ANME-1- and ANME-2-dominant samples (Nauhaus et al., 2005). In both cases, the process was seemingly disconnected from AOM, and we observe no evidence of thiosulfate reductase synthesized during our incubations. The connection between AOM-linked sulfate reduction and thiosulfate metabolism remains uncertain, but may impact sulfur cycling in important ways.

Enzymes facilitating sulfur oxidizing pathways were present in the co-localized metagenomes, indicating that the sediments possess a widespread potential for sulfur compound oxidation. Tag sequencing data revealed a substantial proportion of the *Sulfurovum* genus, accounting for between 4 and 9.6% of total 16S rRNA gene sequences (Supplemental Data Sheet 3a). *Sulfurovum* species are frequently invoked as sulfur-oxidizing constituents in anoxic zones (Sylvan et al., 2012; Schunck et al., 2013; Urich et al., 2014); their high 16S rRNA gene relative abundance, combined with the low recovery of associated sulfur-oxidizing proteins, suggests that DNA signatures of this lineage are retained over the experimental timescale despite the absence of substantial protein-based biosynthesis.

## Conclusion

This study represents one of the most comprehensive SIP proteomics investigations of a complex environmental milieu carried out to date, revealing protein synthesis in slow-growing methane seep sediment communities with a high degree of functional and phylogenetic detail. The detection of multiple functionally relevant orthologs provides a broad sense of *in situ* active metabolic pathways, while the incorporation of stable isotope probing methods reveals the subset of proteins actively produced under laboratory-based methanotrophic conditions. Components of all of the proteins involved in the reverse methanogenesis pathway were identified, and Mcr was shown to account for 10.4% of all detected peptides. The prevalence of ^15^N enriched orthologs involved in reverse methanogenesis and sulfate reducing pathways bolsters our understanding that AOM is the dominant biogeochemical process in seep-simulating incubation conditions, indicating that metabolic activity need not scale with community composition as determined by 16S rRNA gene relative abundances. MS-based PTM identification is well-suited to reveal a wider functional diversity than that encoded by nucleic acid sequences alone, a potentially common reality with significant metabolic repercussions. We were able to detect several enriched, pervasive proteins that lack functional annotations, highlighting the utility of proteomic SIP as a discovery platform for ecologically important proteins that should be prioritized for subsequent biochemical characterization. Finally, proteomic SIP can inform more focused proteomic investigations to quantify enzymes of pathways of interest; such values could then be integrated into flux balance models of carbon, sulfur, and nitrogen cycles to better constrain the dynamics and rates of reactions at both the consortium and ecosystem scales. Similar analyses of methanogenic habitats or cultures would provide additional information on the differences between methanogenic and methanotrophic pathways, potentially revealing activity-based controls on methane-linked metabolism.

Tracking the metabolic activity of energy-limited microbial communities has long challenged analytical techniques that address specific microscale anabolic reactions, system-wide metabolic potential, or broad biogeochemical transformations. Proteomic SIP enables the functionally-, phylogenetically-, and temporally-constrained understanding of protein synthesis, opening access to a previously untapped array of distributed low-growth microorganisms active in selected catabolic pathways. Developing a more nuanced appreciation of metabolite flow and interspecies interaction in this way is crucial for the improved understanding and management of microbial ecosystems and their global impacts.

## Author contributions

JM, CS, RH, and VO designed the study; JM performed incubation set-up, microbiological and geochemical analyses; CS constructed the metagenomic databases and developed the computational architecture for protein analysis; KC developed protein extraction procedures; ZL and CP performed proteomic SIP computational analysis; JM analyzed proteomic findings from an environmental microbiological perspective and wrote the manuscript with input from all authors.

## Funding

This work was supported by the US Department of Energy, Office of Science, Office of Biological Environmental Research, under award numbers DE-SC0004949 and DE-SC0010574, and the Life Underground NASA Astrobiology Institute (NNA13AA92A) (to VO). JM was supported by a National Energy Technology Laboratory Methane Hydrate Research Fellowship funded by the National Research Council of the National Academies. The funders had no role in study design, data collection and interpretation, or the manuscript preparation and submission process.

### Conflict of interest statement

The authors declare that the research was conducted in the absence of any commercial or financial relationships that could be construed as a potential conflict of interest.
