# Supplementary material for: Proteomic Stable Isotope Probing Reveals Biosynthesis Dynamics of Slow Growing Methane Based Microbial Communities
Source: Front Microbiol. 2016 Apr 29;7:563. doi: 10.3389/fmicb.2016.00563 (PMC4850331; doi:10.3389/fmicb.2016.00563)
Supplement: Supplementary file 1 [file Presentation1.PDF]

frontiers

Supplementary Material

Proteomic Stable Isotope Probing Reveals Biosynthesis Dynamics of Slow Growing Methane Based Microbial Communities

Jeffrey J. Marlow*, Connor T. Skennerton, Zhou Li, Karuna Chourey, Robert L. Hettich, Chongle Pan, Victoria J. Orphan*

*** Correspondence:** Corresponding Authors: JJM: marlow@fas.harvard.edu; VJO: vorphan@gps.caltech.edu

1. Supplementary Discussion

McrA Post-Translational Modifications

Twenty-eight PTMs were detected (see main text); none appear to be associated with active-site residues (1), potentially due to the apparent preference for unreactive amino acids in this portion of the enzyme to protect against reactive radical intermediates (2). Among Mcr subunits attributed to ANME, an average of 1 PTM per 84 amino acids was detected. This high rate of occurrence is consistent with previous MS-based analyses of environmental systems: 29% of a dominant constituent's proteins exhibited PTMs in an acid mine drainage biofilm, with distinct modification profiles among closely related organisms (3). Our account may be an under-representation of PTM pervasiveness, as many known but less common modifications were not included in the search space (4) and others may have been under-detected due to incompatible procedural steps (5,6).

PTMs may influence a range of functions, including sensing and signaling (7), formation and activity of protein complexes (8), stability and subcellular localization (9), and protein folding or degradation (10). Methylation was the most common detected McrA modification (Fig. 5), which has been implicated in stress response (11), protein repair (12), and signal transduction (13). The single non-ANME McrA protein, which shows closest homology with cultured *Methanosarcinaceae*, did not exhibit any of the PTMs included in the search parameters (Fig. 5). Only one of the seven previously proposed PTMs (14) was potentially discoverable based on detected peptides and their attendant phylogenic assignments; it was not observed (Fig. 5).

Nitrogen Metabolism

Methane oxidizing environments are intimately linked with nitrogen metabolisms, as revealed through AOM coupled to nitrate and nitrite reduction (15,16) and nitrogen fixation by ANME within consortia (17). Components of all enzymes involved in reductive nitrogen metabolisms such as dissimilatory nitrate reduction, denitrification, and nitrogen fixation are present within the cumulative metaproteome (Fig. S2); in contrast, no nitrogen metabolism enzymes were reported from the Nyegga seep (18). Detected proteins include membrane-bound NarG and NorC, a cytochrome-containing nitric oxide reductase subunit that processes the toxic NO intermediate into N_2O (19). Only one copy of NirB was in the enriched fraction of the #5133 ^{15}N proteome, suggesting that

nitrogen metabolism was not a substantial aspect of community function under incubation conditions. The lack of proteins involved in the assimilatory reductive pathway (NasAB, NarB) suggests that the seep sediment system is not limited for bioavailable nitrogen. Nitrogenase is well represented, with six NifH and two NifK protein orthologs identified across all samples; the three archaeal NifH orthologs that were recovered are consistent with observations of ANME nitrogen fixation (17). Nitrogen fixation is an energetically demanding undertaking, which may explain the lack of enriched Nif proteins generated under conditions of abundant (1mM) bioavailable NH_4^+.

The detection of substantial portions of nitrate reducing, denitrifying, and nitrogen fixing pathways, coupled with the recovery of almost no newly synthesized proteins, suggests that nitrogen-based metabolisms – particularly among *Epsilon*- and *Gammaproteobacteria* – may be important in seep sediments; however, methane- and ammonium-rich incubation conditions rendered such metabolisms unnecessary or energetically untenable.

2. Supplementary Figures and Tables

**Study**	**Sample**	**Protein Identifications**	
		2TP 1UP	1TP 1UP
This Study	*Hydrate Ridge Cold Seep*	*3495*	*5664*
Pan et al., 2011	Acid Mine Drainage biofilm	815-2326	
Denef et al., 2009	Acid Mine Drainage	2752**	
Lo et al., 2007	Acid Mine Drainage	3234	
Ram et al., 2005	Acid Mine Drainage	2033	5090
Stokke et al., 2012	Nyegga Cold Seep	356	
Urich et al., 2014	Trollveggen Hydrothermal Vent Field Microbial Mat	1012	1408*
Dong et al., 2010	South China Sea water column	505	3035
Sowell et al., 2009	Sargasso Sea surface waters		1042
Kan et al., 2005	Chesapeake Bay Estuary	3	
Liu et al., 2012	Sponge symbiont community	765	
Markert et al., 2007	Riftia Symbionts	220	
Benndorf et al., 2007	Contaminated Soil / Groundwater		59
Schulze et al., 2005	Hohloh Lake, Hainich Soil		513
Lacerda et al., 2007	Wastewater Treatment Reactor	109	
Wilmes et al., 2008a	Wastewater Sludge Batch Reactor	46	
Wilmes et al., 2008b	Wastewater Sludge Batch Reactor	2378	
Park et al., 2008	Wastewater Sludge EPS		10
VerBerkmoes et al., 2009	Human Gut	2214	

Table S1: The number of proteins identified in selected previous metaproteomic studies, using a variety of protein extraction, digestion, separation, MS, and search parameters. *1TP, 1UP proteins were only included when corresponding mRNA sequence was present. **Average from 27 samples; 3 MS/MS runs for each sample.

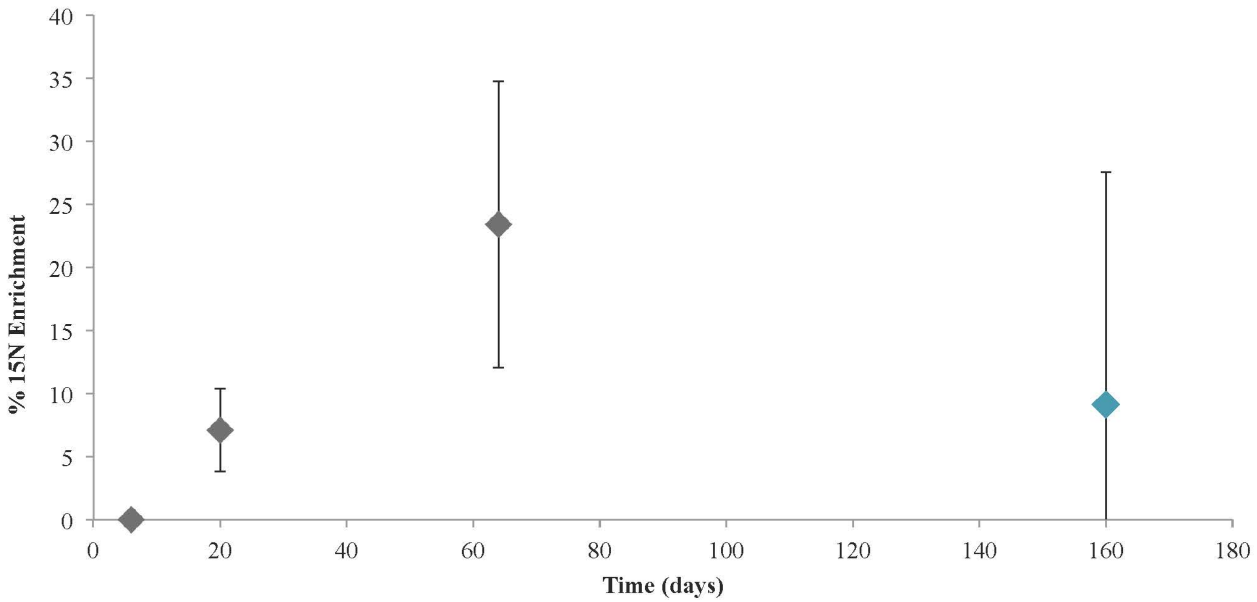

Supplementary Figure 1: ^{15}N enrichment values of whole aggregates analyzed by nanoSIMS (gray diamonds) and proteome-derived peptides (blue diamond) for sample #5133 ^{15}N (whose wide error bars are attributable to the near-binary enrichment distribution shown in Fig. 3). Natural abundance ^{15}N (0.36 atom %, as determined from analysis of Clostridia spores, n=3) was subtracted from all data points. For whole aggregate analysis, N=34 at T=6 d, N=81 at T=20 d, and N=54 at T=64 d.

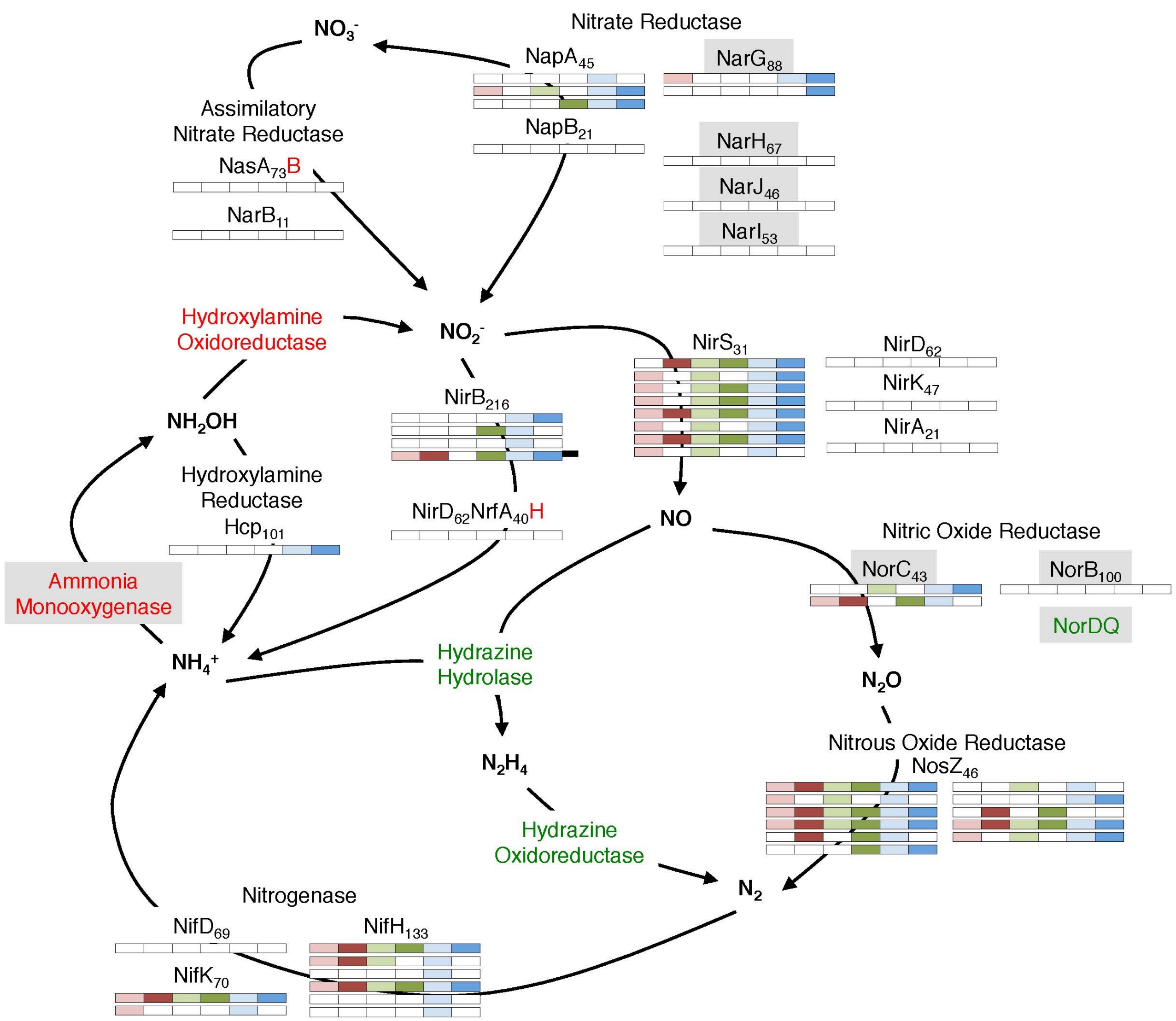

Supplementary Figure 2: Metaproteomic data for enzymes involved in nitrogen metabolism. For key, see Fig. 4.

3. Supplementary Data File Legends

Supplementary Data File 1: The makefile used in this study for processing Illumina MiSeq sequencing data.

Supplementary Data File 2: A table of the cultured organisms retrieved from NCBI whose genomes were incorporated into the metagenomic database.

Supplementary Data File 3: Relative abundance of OTUs identified by 16S rRNA gene tag sequencing (3a) and relative abundance of OTUs associated with methanogens and anaerobic methanotrophs (3b).

Supplementary Data File 4: Sample distributions and phylogenetic assignments of all reverse methanogenesis (4a) and sulfate reduction pathway proteins (4b, 4c) detected in this study.

4. Supplementary References

1. Shima, S. *et al.* Structure of a methyl-coenzyme M reductase from Black Sea mats that oxidize methane anaerobically. *Nature* **481,** 98–101 (2012).
2. Ermler, U., Grabarse, W., Shima, S., Goubeaud, M. & Thauer, R. K. Crystal Structure of Methyl-Coenzyme M Reductase: The Key Enzyme of Biological Methane Formation. *Science* **278,** 1457–1462 (1997).
3. Li, Z. *et al.* Diverse and divergent protein post-translational modifications in two growth stages of a natural microbial community. *Nat Commun* **5,** (2014).
4. Wang, Y., Ahn, T.-H., Li, Z. & Pan, C. Sipros/ProRata: a versatile informatics system for quantitative community proteomics. *Bioinformatics* **29,** 2064–2065 (2013).
5. Gilmore, J. M., Kettenbach, A. N. & Gerber, S. A. Increasing phosphoproteomic coverage through sequential digestion by complementary proteases. *Analytical and bioanalytical chemistry* **402,** 711–720 (2012).
6. Olsen, J. V. & Mann, M. Status of large-scale analysis of post-translational modifications by mass spectrometry. *Molecular & Cellular Proteomics* **12,** 3444–3452 (2013).
7. Ivan, M. *et al.* HIFα Targeted for VHL-Mediated Destruction by Proline Hydroxylation: Implications for O2 Sensing. *Science* **292,** 464–468 (2001).
8. Kovacs, J. J. *et al.* HDAC6 Regulates Hsp90 Acetylation and Chaperone-Dependent Activation of Glucocorticoid Receptor. *Molecular Cell* **18,** 601–607 (2005).
9. Chuikov, S. *et al.* Regulation of p53 activity through lysine methylation. *Nature* **432,** 353–360 (2004).
10. Deribe, Y. L., Pawson, T. & Dikic, I. Post-translational modifications in signal integration. *Nat Struct Mol Biol* **17,** 666–672 (2010).
11. Desrosiers, R. & Tanguay, R. Methylation of Drosophila histones at proline, lysine, and arginine residues during heat shock. *Journal of Biological Chemistry* **263,** 4686–4692 (1988).
12. Najbauer, J., Orpiszewski, J. & Aswad, D. W. Molecular Aging of Tubulin: Accumulation of Isoaspartyl Sites in Vitro and in Vivo. *Biochemistry* **35,** 5183–5190 (1996).
13. Aletta, J. M., Cimato, T. R. & Ettinger, M. J. Protein methylation: a signal event in post-translational modification. *Trends in Biochemical Sciences* **23,** 89–91 (1998).

14. Kahnt, J. *et al.* Post-translational modifications in the active site region of methyl-coenzyme M reductase from methanogenic and methanotrophic archaea. *FEBS Journal* **274,** 4913–4921 (2007).

15. Raghoebarsing, A. A. *et al.* A microbial consortium couples anaerobic methane oxidation to denitrification. *Nature* **440,** 918–921 (2006).

16. Haroon, M. F. *et al.* Anaerobic oxidation of methane coupled to nitrate reduction in a novel archaeal lineage. *Nature* **500,** 567–570 (2013).

17. Dekas, A., Poretsky, R. S. & Orphan, V. J. Deep-Sea Archaea Fix and Share Nitrogen in Methane-Consuming Microbial Consortia. *Science* **326,** 422–426 (2009).

18. Stokke, R., Roalkvam, I., Lanzen, A., Haflidason, H. & Steen, I. H. Integrated metagenomic and metaproteomic analyses of an ANME-1-dominated community in marine cold seep sediments. *Environmental microbiology* **14,** 1333–1346 (2012).

19. Mesa, S., Velasco, L., Manzanera, M. E., Delgado, M. J. & Bedmar, E. J. Characterization of the norCBQD genes, encoding nitric oxide reductase, in the nitrogen fixing bacterium Bradyrhizobium japonicum. *Microbiology* **148,** 3553–3560 (2002).
