# Supplementary material for: Proteomic Stable Isotope Probing Reveals Biosynthesis Dynamics of Slow Growing Methane Based Microbial Communities
Source: Front Microbiol. 2016 Apr 29;7:563. doi: 10.3389/fmicb.2016.00563 (PMC4850331; doi:10.3389/fmicb.2016.00563)
Supplement: Supplementary file 2 [file DataSheet1.pdf]

```
#!/usr/bin/make -f

SHELL = /bin/bash

R1_FILES_GZ = $(wildcard *_R1_001.fastq.gz)
R1_FILES = $(basename $(R1_FILES_GZ))
R2_FILES_GZ = $(wildcard *_R2_001.fastq.gz)
R2_FILES = $(basename $(R2_FILES_GZ))

SSU_DB = /export/data1/db/16S_tag_processing_db/
SSURef_NR99_Silva_115_pintailF_ORPHAN.fasta
SSU_TAX = /export/data1/db/16S_tag_processing_db/
SSURef_NR99_Silva_115_pintailF_ORPHAN.tax
USEARCH = usearch
CHIMERA = 0

qiime_programs = pick_otus.py pick_rep_set.py assign_taxonomy.py
make_otu_table.py filter_otus_from_otu_table.py summarize_taxa.py
join_paired_ends.py split_libraries_fastq.py

.PHONY: help clean all check_dependancies

help:
	@echo "This is a makefile containing the standard Orphan Lab
protocol for processing tag data" \
		"This makefile should be placed in a new directory
containing just the fastq files produced" \
			"from the samples that you wish to analyse. You can
achieve this by either copying the files with the cp command" \
			"into this directory or by creating links
(shortcuts) using the ln command." \
			"To run the analysis you need to have the qiime" \
			"module loaded into your path. type the following
into the command line:" | fmt
	@echo
	@echo "module load qiime"
	@echo
	@echo "The pipeline can be run using one of the following
commands:"
	@echo
	@echo "./orphanlab_itag_protocol.mk all"
	@echo "make -f orphanlab_itag_protocol.mk all"
	@echo
	@echo "By default, no chimera checking takes place. However
you can add this as an option to the pipeline" \
			" by specifying CHIMERA=1 on the commandline like
so:" | fmt
	@echo
	@echo "./orphanlab_itag_protocol.mk CHIMERA=1 all"
	@echo
```

```
	@echo "When chimera checking is turned on, usearch is an added
dependancy. Make sure it is in your path by typing:" | fmt
	@echo
	@echo "module load usearch"
	@echo
	@echo "The files for taxonomic assignment used by this
pipeline are currently:"
	@echo $(SSU_DB)
	@echo $(SSU_TAX)
	@echo
	@echo "If you would like to use other files, type the
following into the command line:"
	@echo
	@echo "./orphanlab_itag_protocol.mk all SSU_DB=\"/path/to/
custom/fasta_file\" SSU_TAX=\"/path/to/custom/taxonomy\""
	@echo
	@echo "You should always specify both as they need to match"

check_dependancies:
	@if [ ! -e "$(SSU_DB)" ]; then echo "could not find the file $
(SSU_DB). Please verify that the path is correct or add a custom
location (type: "make help" for more details)"; exit 1; fi
	@if [ ! -e "$(SSU_TAX)" ]; then echo "could not find the file
$(SSU_TAX). Please verify that the path is correct or add a custom
location (type: "make help" for more details)"; exit 1; fi
	@for i in $(qiime_programs); do which $$i &>/dev/null; if [ !
$$? -eq 0 ]; then echo "could not find $$i in your path. you probably
don't have qiime installed/loaded correctly"; exit 1;fi  done
ifneq ($(CHIMERA),0)
	@which $(USEARCH) &>/dev/null; if [ ! $$? -eq 0 ]; then echo
"could not find usearch in your path. Please install it"; exit 1; fi
endif


all: check_dependancies $(R1_FILES) $(R2_FILES)
uclust_taxa_0.9_10_0.90/singletonfiltered_taxa_summary

clean:
	-rm -r *joined *trimmed *chimerachecked

make_dummy_mapping.pl: $(R1_FILES)
	echo 'my @files; my $$index; my @words = split / /, `echo
{A,C,T,G}{A,C,T,G}{A,C,T,G}{A,C,T,G}{A,C,T,G}{A,C,T,G}{A,C,T,G}`;
while (<>) {   chomp;  @files = split;  for($$index=0;$$index< scalar
@files; $$index++) {  $$files[$$index] =~ s/_S\d+_L\d+_R1_\d+\.fastq$
$//; $$files[$$index] =~ s/-/./g; print "$$files[$$index]\t$$words[$
$index]\tA\tA\tGeneric\n"; }  }  ' > $@

dummy_mapping2.txt:
	echo "#SampleID  BarcodeSequence  LinkerPrimerSequence
```

```
ReversePrimer    Description" > $@
        echo -e "A\tA\tA\tA\tA" >> $@

dummy_mapping.txt: make_dummy_mapping.pl
        echo "#SampleID  BarcodeSequence  LinkerPrimerSequence
ReversePrimer    Description" > $@
        echo $(R1_FILES) | perl $< >> $@

uclust_picked_otus_99/all_seqs_all_samples_otus.txt:
all_seqs_all_samples.fna
        pick_otus.py -i $< -s 0.99 -o $(@D)

all_seqs_all_samples.fna_rep_set.fasta: all_seqs_all_samples.fna
uclust_picked_otus_99/all_seqs_all_samples_otus.txt
        pick_rep_set.py -i $(word 2,$^) -f $< -m most_abundant

uclust_taxa_0.9_10_0.90/
all_seqs_all_samples.fna_rep_set_tax_assignments.txt:
all_seqs_all_samples.fna_rep_set.fasta
        assign_taxonomy.py -i $< \
                -t $(SSU_TAX) \
                -r $(SSU_DB) \
                --uclust_similarity 0.9 \
                --uclust_max_accepts 10 \
                --uclust_min_consensus_fraction 0.90 \
                -o $(@D)

uclust_taxa_0.9_10_0.90/OTU_table_Silva_115_all_seqs.biom:
uclust_picked_otus_99/all_seqs_all_samples_otus.txt
uclust_taxa_0.9_10_0.90/
all_seqs_all_samples.fna_rep_set_tax_assignments.txt
        make_otu_table.py -i $< -t $(word 2, $^) -o $@

uclust_taxa_0.9_10_0.90/OTU_table_singletonfiltered.biom:
uclust_taxa_0.9_10_0.90/OTU_table_Silva_115_all_seqs.biom
        filter_otus_from_otu_table.py -i $< -n 2 -o $@

uclust_taxa_0.9_10_0.90/singletonfiltered_taxa_summary:
uclust_taxa_0.9_10_0.90/OTU_table_singletonfiltered.biom
        summarize_taxa.py -i $< -a -o $@

%: %.gz
        gunzip -c $< >$@

%.fastq_joined/fastqjoin.join.fastq: %.fastq
        join_paired_ends.py -f $< -r $(subst _R1_,_R2_,$<) -m fastq-
join -j 50 -p 8 -o $(@D)

%.fastq_trimmed/seqs.fna: %.fastq_joined/fastqjoin.join.fastq
dummy_mapping2.txt
```

```
        split_libraries_fastq.py -i $< -o $(@D) -m $(word 2,$^) --
sample_id $* -q 29 -n 0 --barcode_type 'not-barcoded' --
store_qual_scores

%.fastq_chimerachecked/uchime_nonchimeras.fna: %.fastq_trimmed/
seqs.fna $(SSU_DB)
        -mkdir $(@D)
        $(USEARCH) -uchime_ref $< \
                -db $(word 2,$^) \
                -uchimeout $*.fastq_chimerachecked/results.uchime \
                --strand plus \
                -chimeras $*.fastq_chimerachecked/uchime_chimeras.fna
\
                -nonchimeras $@

ifeq ($(CHIMERA),0)
all_seqs_all_samples.fna: $(addsuffix _trimmed/seqs.fna, $(R1_FILES))
        cat $^ >$@
else
all_seqs_all_samples.fna: $(addsuffix _chimerachecked/
uchime_nonchimeras.fna, $(R1_FILES))
        cat $^ >$@
endif
```
